# Accelerating Expertise: A Fast-Track Training Program for Living Lab Methodologies

**DOI:** 10.12688/openreseurope.21200.1

**Published:** 2025-11-10

**Authors:** Kim Helsen, Vicky Van der Auwera, Femke Drijkoningen, Despoina Petsani, Teemu Santonen, Panagiotis Bamidis, Evdokimos Konstantinidis, Nele A.J. De Witte

**Affiliations:** 1 LiCalab Living & Care Lab, Centre of Expertise Care and Well-being, Thomas More University of Applied Sciences, Geel, 2440, Belgium; 2 Medical Physics and Digital Innovation Laboratory, School of Medicine, Aristotle University of Thessaloniki, Thessaloniki, Greece; 3 Laurea University of Applied Sciences, Vantaa, Finland

**Keywords:** Training, user-centred design, Living Lab, participatory design, education, harmonisation, methodology

## Abstract

**Background:**

Living Labs have proven to be valuable environments for fostering innovation through user-centred approaches. However, many researchers and companies still face challenges in implementing these methodologies sustainably. Addressing these challenges requires not only structural solutions within Living Labs, but also the cultivation of expertise among researchers and practitioners. It is crucial to educate researchers and innovators in practices aligned with user-centred research, living lab practices and co-design, emphasizing societal relevance and Responsible Research and Innovation (RRI) within the research community.

**Method:**

This paper presents and evaluates a novel training program developed within the VITALISE project, aimed at onboarding external researchers and familiarizing them with Living Lab Research Infrastructures through transnational visits and collaborations.

**Results:**

The program features a modular design covering key topics such as Living Lab methodology, harmonization of research practices, and participant recruitment and panel management. A total of 49 participants completed an evaluation questionnaire, with results indicating high satisfaction and perceived usefulness across all training modules. Post-training, most participants reported increased confidence in applying Living Lab methodologies. Notably, individual differences in interest across training blocks highlighted the need for flexible, tailored programs that accommodate varying levels of prior knowledge and specific research needs.

**Conclusion:**

This study suggests that targeted, adaptable training initiatives are acceptable and can help to enable researchers to integrate Living Lab methodologies into their work. Continued development of structured, scalable, and context-sensitive training programs, supported by international collaborations and standardized approaches, will be essential for fostering sustainable and impactful Living Lab research across disciplines and borders.

## Plain Language Summary

Living Labs are environments where researchers, companies, and citizens work together to test and improve new ideas and technologies in real-life settings. Although these labs can help create better and more useful innovations, many researchers still struggle to use such environments (or the methods they use) effectively. To help with this, a European project called VITALISE created a training program to teach researchers about living labs and the methods they use. The training included different topics like how to involve people in research, how to manage participants, and how to make research methods more consistent across labs. Forty-nine researchers took part in an evaluation of the training and gave feedback. Most of them were very satisfied and felt more confident using Living Lab methods afterward. The training was designed to be flexible, so people with different backgrounds and needs could benefit from it. This study shows that well-designed training programs can help researchers use Living Labs more successfully, which could lead to better innovations that are more connected to society’s needs.

## Introduction

Over the last decades, Living Labs have emerged as resilient research infrastructures and facilitate the transition from research outcomes to market implementation ( Fauth *et al.*, 2024; Leminen & Westerlund, 2016). Living Labs can be defined as open innovation ecosystems in real-life operational environments which put citizens and/or end-users at the centre of the innovation process ( European Network of Living Labs, 2025). They promote a systematic user co-creation approach that integrates research and innovation activities in communities and/or multi-stakeholder environments. Beyond their role as co-creation environments, Living Labs act as orchestrators within regional innovation ecosystems, facilitating collaboration across stakeholders and aligning innovation activities with societal challenges ( Fauth *et al.*, 2024). These ecosystems reflect the principles of the Quadruple Helix innovation model, where collaboration between academia, industry, government, and civil society drives knowledge creation and sustainable innovation ( Carayannis & Campbell, 2010). In the Health and Wellbeing domain, where the needs of people such as young individuals, older adults, and patients are central, this approach is particularly valuable for creating meaningful, context-sensitive solutions.

Living Labs aim not only to explore users’ contexts, behaviours, and preferences but also to foster co-creation processes that generate societal value and engage diverse stakeholders ( Westerlund *et al.*, 2018). Co-creation, however, extends beyond a methodological approach; it represents a mindset rooted in participatory design, emphasizing the belief that all individuals can meaningfully and creatively contribute to innovation processes when equipped with the right tools and frameworks ( Sanders & Stappers, 2008). This vision aligns closely with broader movements in participatory health research and human-centered innovation, where collaborative design with stakeholders is key to achieving sustainable impact.

Despite their potential, Living Labs face significant challenges. These include navigating complex governance structures, engaging and retaining stakeholders, and addressing obstacles in executing co-creation activities and real-world pilot testing. Barriers range from designing appropriate study protocols and ensuring user engagement to tackling ethical dilemmas and technical implementation issues ( Fauth *et al.*, 2024). Furthermore, the field is hindered by issues of sustainability ( Fauth *et al.*, 2024; Paskaleva & Cooper, 2021) and a lack of harmonized services, methods, and terminology ( Santonen *et al.*, 2024b). Particularly in the Health and Wellbeing domain, the diversity of digital data collection and intervention tools, ranging from wearables to mobile applications and sensor-based platforms, reflects the methodological heterogeneity that characterizes many Living Lab activities ( Petsani *et al.*, 2024). This methodological diversity and complexity also impede the systematic documentation of effectiveness and the transparent reporting of outcomes ( Paskaleva & Cooper, 2021).

Addressing these challenges requires not only structural solutions within Living Labs, but also the cultivation of expertise among researchers and practitioners. Establishing and conducting high-quality co-creative processes requires specialized expertise and considerable effort from both academic researchers ( De Silva *et al.*, 2023) and small and medium-sized enterprises (SMEs). Access to Living Lab infrastructures alone is insufficient; researchers and organizations must also develop the necessary skills and understanding to apply Living Lab methodologies effectively and consistently.

Education plays a critical role in building these competencies. To this end, it is essential to educate new generations of researchers in user-centered approaches, fostering a strong societal orientation in line with the principles of Responsible Research and Innovation (RRI). RRI emphasizes the need for inclusive, anticipatory, and reflexive innovation practices that align research activities with societal values and needs ( Owen & Pansera, 2019). In the context of Living Labs, this implies embedding societal stakeholders meaningfully into research and innovation processes, not only as users but as co-creators of knowledge ( Van Geenhuizen, 2019). User experience and participatory design have for some years been integrated into the curricula of students in fields such as engineering, product development, and human-computer interaction, and can go beyond theory to include experiential learning ( Hecht & Maass, 2008; Kang *et al.*, 2022). However, students in healthcare disciplines (e.g., nursing, healthcare management, occupational therapy) are rarely exposed to these methodologies. Therefore, training initiatives must extend beyond student education to include active researchers and practitioners from diverse academic and commercial backgrounds. Notably, a study by Konstantinidis *et al.* (2021) demonstrated that even brief training interventions (e.g., an 8-hour program) can significantly enhance participants’ understanding of co-creative methods, as evidenced in the case of engineering students. This highlights the value of targeted training for fostering co-creation competencies across disciplines.

One initiative that sought to address this need for capacity building and harmonization in the Living Lab community is the European Horizon 2020 project VITALISE. This project aimed to enhance and promote research in the Health and Wellbeing domain by opening access to Living Lab infrastructures and services across Europe and beyond. Through VITALISE, researchers could obtain in-person Transnational Access to 17 Living Lab research infrastructures, supporting studies in areas such as rehabilitation, transitional care, and everyday life environments. In these collaborative settings involving Living Labs, incorporating a training component on Living Lab methodologies can be highly beneficial. Integrating training with active research collaboration not only enhances learning through experiential engagement ( Kang *et al.*, 2022), but also supports the harmonization and interoperability of Living Lab practices across diverse initiatives and ecosystems ( Santonen *et al.*, 2024b).

This paper describes how the VITALISE project developed and implemented a fast-track-training method to broaden the understanding and valorisation of Living Lab methodologies within the research community. Specifically, we present the design and content of this training program and report on its evaluation through a participant survey.

## Method

### Research design framework

**Figure 1.**
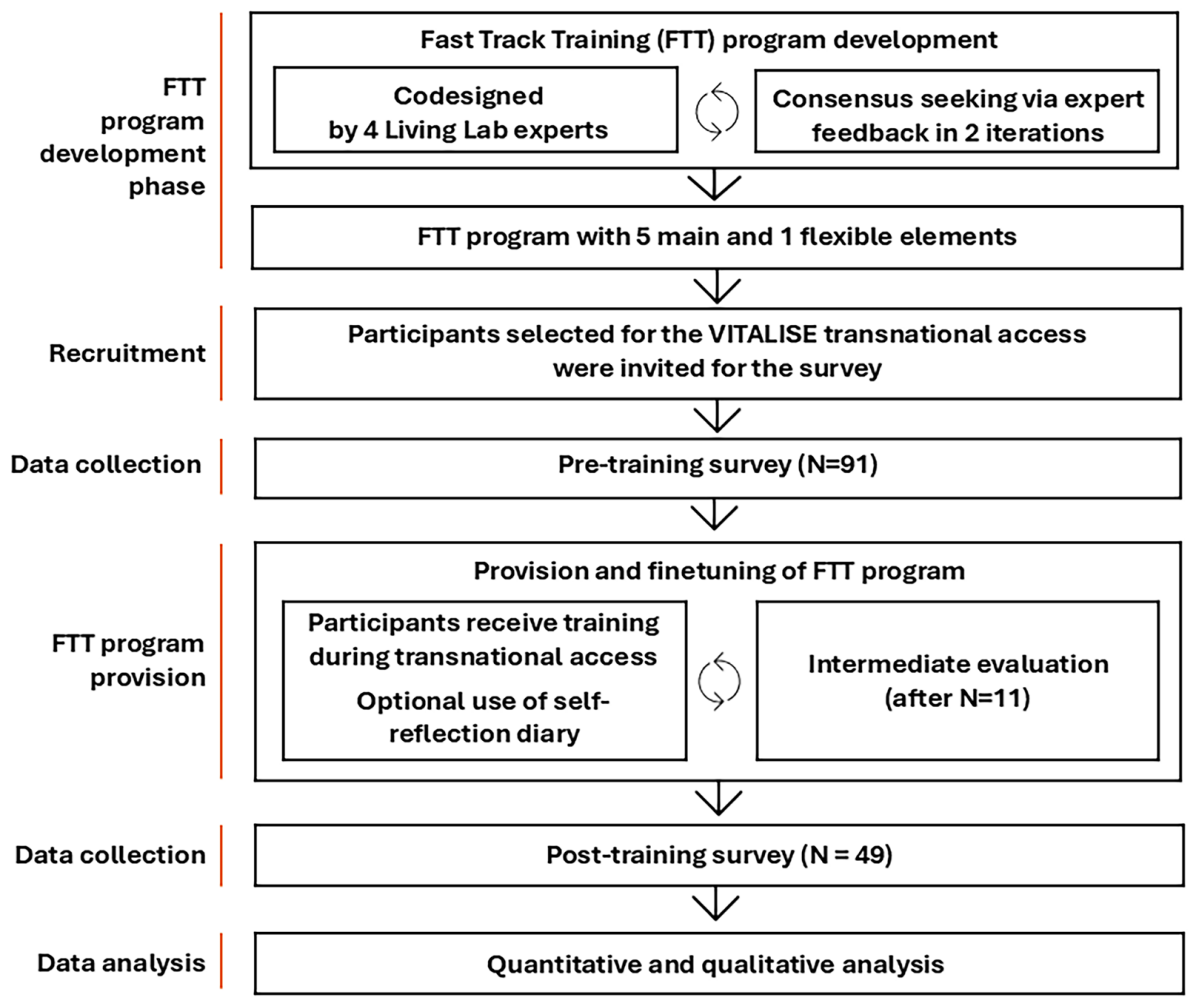
Overview of Research Phases.

Figure 1 presents the six sequential phases of the study. An iterative, consensus-seeking approach, conducted in collaboration with a group of experts, was employed to develop the Fast Track Training (FTT) programme, which comprises five core elements and one flexible component. An open call for participation in transnational access, including the training, was organised. Eligibility criteria were applied to select participants, who were then invited to complete a pre-training survey. At the beginning of the transnational visit, participants received training and were introduced to the use of a self-reflection diary. An intermediate analysis, conducted after the visits of the first 11 researchers, was undertaken to evaluate and refine the FTT programme based on real-life experiences. A post-training evaluation questionnaire was administered upon completion of the programme. Finally, quantitative and qualitative analyses were carried out using data from the pre- and post-training surveys. The following sections describe each phase in more detail.

### Fast Track Training (FTT) program development

The training program was constructed under the lead of LiCalab Living and Care Lab of Thomas More University of Applied Sciences, a Belgian living lab with over 10 years of experience in Living Lab methodology in the field of health and care. Four senior living lab consultants codesigned a program to immerse researchers in Living Lab methodology during a 2-day training. Next, feedback from all VITALISE project partners on the outline of the program was collected. After consensus was achieved for the topics, the content was built. Five main program elements were selected in addition to one flexible training block in which each hosting living lab was free to add and share an additional topic within their expertise. Once again, input and feedback from all project partners was sought. An intermediate analysis after the visits of the first 11 researchers to varying living labs showed that the need existed to implement the training in a more flexible and tailored way. Additionally it was suggested to focus most on the topics that were of greatest relevance for the planned studies or the topics in which researchers lacked knowledge.

Throughout training and transnational access activities, a self-reflection diary (NoteTheBook) was available. This is a platform developed specifically within the context of VITALISE by one of the consortium partners (TREBAG). The overarching idea behind the NoteTheBook platform is that interacting with the study material by means of taking notes, highlighting texts, sorting out ideas and concepts, marking paragraphs, rewriting sentences in our own words is one of the most commonly and most effectively used techniques for learning. The platform is available at this link: https://vitalise.trebag.hu/. By the end of the Transnational access, researchers would have their own personalised PDF booklet to take home.

### Participant recruitment and selection

The study took place in the context of the VITALISE Transnational Access activities. These activities provided international researchers with the opportunity to access Living Lab infrastructure and receive support in conducting research. The call to participate in the VITALISE TA activities was spread through websites, social media channels and newsletters of VITALISE and the VITALISE project partners, including the European Network for Living Labs (ENoLL). Researchers were eligible to participate in the transnational access if they were employed by institutions in EU Member States or countries associated with the H2020 Programme (but these could not exceed 20% of the total sample). Exclusion criteria were (1) Russian or Belarusian origin of researchers, (2) employment in the same country as the selected living lab, (3) lack of English proficiency (necessary for effective communication with the living labs), (4) master student application without a team member with a master’s degree, (5) undergraduate student without the supervision of a professor or postdoc researcher. Research teams could apply to visit multiple Living Labs, submitting separate applications for each and providing justification for their choices. The living labs participants could visit were Austrian Institute of Technology (AIT), Aristotle University of Thessaloniki (AUTH), Association of Electronic and Information Technologies in the Basque Country (GAIA), INTRAS Foundation (INTRAS), Laurea University of Applied Sciences (Laurea), LiCalab - Living & Care lab at Thomas More University of Applied Sciences (LiCalab), McGill University (McGill), TREBAG Intellectual Property and Project Manager (TREBAG), and Universidad Politécnica de Madrid (UPM). All participants whose application for the call was accepted, were offered the Fast Track Training (FTT). These participants were invited to complete an FTT expectations and FTT evaluation questionnaire. Prior to the Transnational Access (including the training and questionnaires), all participants were presented with a service agreement detailing the expectations from project partners and visitors. The agreement was signed by the project lead and the visiting researchers, hereby providing active written informed consent for participation in the FTT questionnaires.

### Pre- and post- survey questionnaires

Before participating in the Transnational Access and the FTT, participants were asked to indicate which of the 5 topics of the FTT they considered most relevant for them or their team (“During Transnational Access, a 2-day Fast Track Training program is provided comprising the following topics. Which topics do you consider most relevant to you/your team (Multiple answers possible)?”). A second survey was conducted after completing the FTT to evaluate its content. The survey questions can be consulted at Helsen *et al.* (2025). Surveys consisted of multiple choice questions, rating scales, and open-ended questions. The topics of the evaluation questionnaire included satisfaction with the training as a whole, a quality and usefulness evaluation for the fixed training components, and confidence in applying the Living Lab concepts and methodologies in the researcher’s context after the training. Participants were also asked which topic they would not attend again. Multiple answers could be indicated, but they were forced to choose at least one topic to avoid the socially desirable answer ‘none of the above’. The questionnaire also included open question on topics they would like to add and the most appreciated elements of the training.

### Training and data collection procedure

After (teams of) researchers were selected for the transnational access for one of the living labs of the vitalise project, they signed a service agreement including written informed consent for completing the questionnaires. Next, a short questionnaire was sent to the researchers to monitor expectations and help prepare the agenda for the research stay. The schedule of their visit to the living lab infrastructure was prepared. The training was planned and prepared by the local living labs, based on the materials provided by the lead program developer. The program could include a welcoming session, five main blocks and one optional block. However, an abbreviated program could also be provided if some elements were not deemed necessary based on the profile, interests and expectations of the visiting researchers. The training was offered during the transnational access visit or online. An evaluation questionnaire was provided after completion of the program. Some participants only completed the expectations survey. Potential reasons were not attending the transnational access or training and not responding to the survey invitations.

### Data analysis

SPSS 28.0 was used for calculating averages for the ratings provided by the participants. A Friedman Test (with follow-up Wilcoxon Signed Ranks Test) was used to investigate differences between the rating for the training components. Since not all training blocks were provided to all participants, some responses are based on a subsample of respondents. Open ended questions with qualitative data were organised and grouped using thematic and content analyses. The initial thematic analysis was conducted by the first author. After gaining familiarity with the data through a thorough review of the responses to understand their context and tone, the recurring responses were identified, tagged, and grouped where possible. A second researcher subsequently assessed the alignment of the responses with the identified themes and proposed modifications or additional themes where appropriate, in order to reduce potential bias.

## Results

### The Fast-Track Training program (FTT)

**Figure 2.**
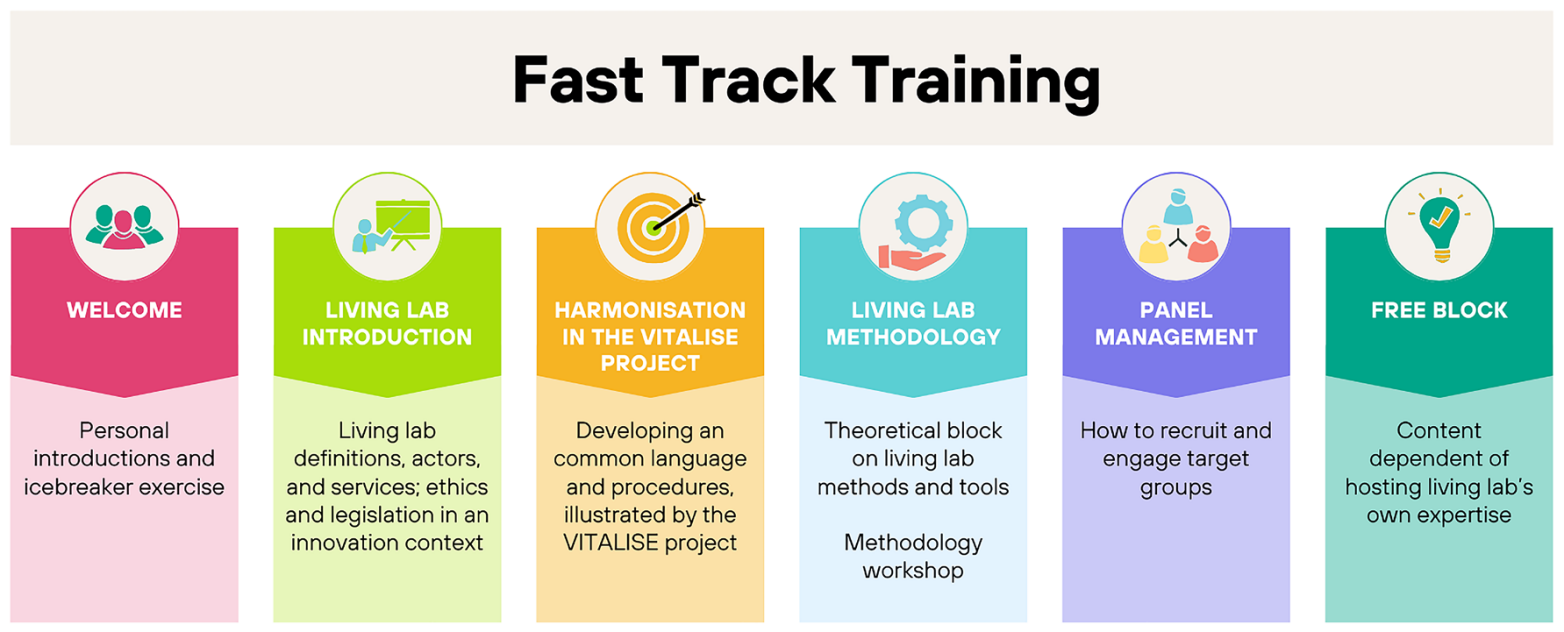
Overview of the training elements.

The full FTT program was structured into six blocks ( Figure 2). The introductory segment lasted approximately one hour. The methodology block was divided into two parts with a combined duration of 2 hours and 30 minutes. The remaining blocks were completed in roughly 1 hour and 30 minutes. The program could be tailored to the needs of the visiting researcher, which implies that not all blocks were presented to all participants.

In the **welcoming part**, both the visiting researchers and the hosting living labs introduced themselves. An icebreaker exercise helped to set the tone. The icebreaker is an animation technique that makes participants more conducive to creativity and sharing. Typically carried out at the start of a meeting, it is an exercise that aims to relax the atmosphere and put the participants in a state of mind of openness necessary for the creative posture.

The second block aimed to provide a broad **introduction in the world of Living Labs**. The participants discovered for example what defines a Living Lab, which roles and actors can be found in the Living Lab context, and which services could be provided. Another important topic in this block was the ethical framework which had proven to be increasingly important in an innovation context where products, contexts and legislation can change at a quick pace. The block concluded with the discussion of ethical challenges and how to tackle them.

The third block was about the **importance of harmonisation**, which is the core business of the VITALISE project. Living Labs can use a variety of procedures operationalised in different ways. A harmonisation of the implemented services, infrastructures and language can help to achieve more cost efficiency, optimal use of time and resources and wider exploitation of research results from local communities to offer their valuable services in the Research and Development community. Therefore, the instructors guided the researchers through the objectives, challenges and different work packages of the VITALISE project, putting the focus on the different joint research activities to be conducted throughout the project.

 The second training day started with a **theoretical block on methodologies**, followed by a methodology workshop. Starting from a design thinking perspective, different methods and tools were discussed. In the sixth block, theory was put into practice with a methodology workshop. Three options were possible: 1) The hosting living lab supported the visiting researchers with further elaboration of their study to be conducted during the visit, 2) the hosting living lab demonstrated a method or tool of their choice, or 3) The UX design speed testing template, provided by one of the consortium partners, was used as an example.

The fifth block comprised the issue of **panel management** and how to engage target groups. Recruitment strategies were examined as well as the motivators and barriers to participate for users. The benefit of having a panel database was explained. Trainers elaborated on how to start building up a panel and how to engage users on a long term. Finally, it also included how to monitor your panel activities.

The **final block** is a free block with content depending on the hosting living lab’s own expertise or best practices. As an example, a training block on how to work with companies and business model preparation was included in Belgium.

The complete set of educational materials prepared for the VITALISE Fast Track Training is also publicly available in the project website: https://vitalise-project.eu/fast-track-training/.

### FTT survey results

***Expectations***

A total of 91 researchers who planned to visit nine different living labs (AIT, *N* = 5; AUTH, *N* = 17; GAIA, *N* = 9; INTRAS, *N* = 14; Laurea, *N* = 11; LiCalab, *N* = 18; McGill, *N* = 4; TREBAG, *N* = 8; and UPM, *N* = 6) completed the expectations questionnaire. Table 1 presents the number and proportion of participants who deemed a proposed training block relevant. The most frequently selected topic was the introduction and ethical framework, followed by target groups and panel management. The harmonization block was selected least often.

**Table 1.** Overview of the frequency and proportion of participants who indicated a certain training block as relevant before receiving the training (multiple blocks could be selected; N = 91).

Topic	N	Proportion (%)
Introduction to Living Labs – Living Lab services – Ethical framework	66	72.53%
Target groups – panel management	47	51.65%
Methodologies workshop	42	46.15%
Methodologies (theoretical framework)	40	43.96%
The importance of harmonisation – VITALISE project	36	39.56%

***Satisfaction and quality of the training***

In total, 49 participants from 17 countries and four professional domains completed the FTT evaluation survey following their visits to seven different living labs (AIT, *N* = 1; AUTH, *N* = 14; GAIA, *N* = 7; INTRAS, *N* = 8; LiCalab, *N* = 7; TREBAG, *N* = 7; and UPM, *N* = 5). More female (N = 31) than male (N = 18) participants responded to the questionnaire and participants originated from many different countries, specifically from the UK (N = 7), Albania (N = 6), Poland (N = 6), Italy (N = 5), Romania (N = 5), France (N = 3), Slovenia (N = 3), Portugal (N = 2), Cyprus (N = 2), Ireland (N = 2), Israël (N = 2), the Netherlands (N = 1), Argentina (N = 1), Norway (N = 1), Belgium (N = 1), Colombia (N = 1), and Spain (N = 1). The majority of participants were from the academic field ( Table 2).

**Table 2.** Researchers’ background.

Domain	N	Proportion (%)
Academia	41	83.67
Entrepreneur	2	4.08
Government researcher	3	6.12
Industrial researcher	2	4.08
Other	1	2.04
**Total**	**49**	

The **overall satisfaction score** regarding the FTT was 9.11 out of a possible 10 ( *SD* = 1.07, Range 5-10; *N* = 47). Almost half of the participants indicated the maximum score ( *N* = 22). Furthermore, we asked researchers to what extent the FTT met their expectations. On a scale from -2 (much worse than expected) to +2 (much better than expected), the mean score was 1.29 ( *SD* = 0.74; *N* = 49). All participants indicated that the FTT was as expected or (much) better.

Researchers rated all **training blocks in terms of quality and usefulness** on a scale of 1 (very low to none) to 5 (very high) ( Table 3). The training blocks were generally deemed of high quality. A Friedman test indicated a significant difference in quality rating between blocks, *χ ^2^* (4, N = 39) = 9.495, *p* = 0.05. A Wilcoxon signed-rank test indicated that there was only a significant difference between the Methodology – Theory block and the Harmonisation block, *z* = -2.829, *p* = .005. The Methodology – Theory block was rated significantly higher than the Harmonisation block. The average usefulness of all training blocks was also high, with minor differences indicating the highest usefulness of *Panel management* and lowest for *The Vitalise project and the importance of harmonisation*, although opinions on this topic varied widely. The Friedman test showed that the differences between the usefulness of the blocks did not reach significance, *χ ^2^* (4, N = 39) = 8.642, *p* = 0.07, although the result suggested a trend towards significance. The result of the analysis presented in Table 3 can also be found at ( Konstantinidis *et al.*, 2025).

**Table 3.** Quality and usefulness rating of the Fast Track Training blocks.

		Quality ^1^					Usefulness ^2^			
Training block	N	Mean	SD	Min	Max	N	Mean	SD	Min	Max
Living Lab introduction	48	4.63	.64	3	5	48	4.50	.77	3	5
Harmonisation in the VITALISE project	48	4.46	.65	3	5	47	4.21	.93	2	5
Living Lab methodology - Theory	46	4.76	.43	4	5	45	4.58	.66	3	5
Living Lab methodology - Workshop	41	4.71	.60	2	5	42	4.55	.63	3	5
Panel management	44	4.61	.69	2	5	44	4.68	.52	3	5

*Note.* ^1^ Quality rating scale: 1 very low quality, 2 low quality, 3 medium quality, 4 high quality, 5 very high quality – not applicable. ^2^ Usefulness rating scale: 1 no usefulness, 2 low usefulness, 3 medium usefulness, 4 high usefulness, 5 very high usefulness, not applicable.

**Figure 3.**
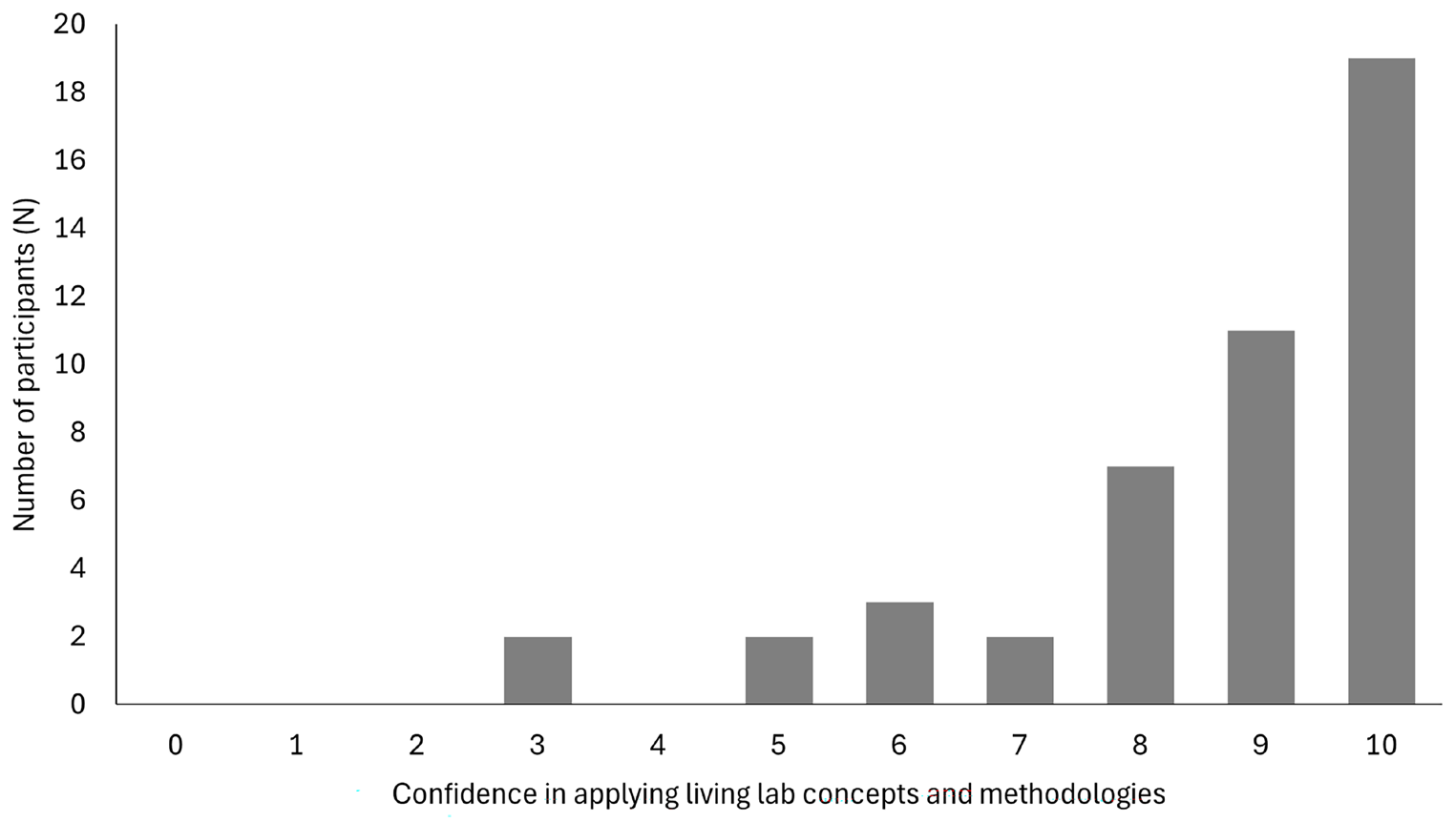
Confidence in applying the Living Lab concepts and methodology in their specific context after training (0 not at all - 10 very confident).

Forty-six participants (46) responded to the question how confident they felt after the FTT in applying the Living Lab concepts and methodology in their own specific context, on a scale from 0 (not at all) to 10 (very confident). Most participants were positive with a mean of score 8.54 ( *SD* = 1.86) ( Figure 3).

***Least appreciated training block***

Participants were asked which of the training blocks they would not like to attend again (forced choice). If they needed to cut one block, almost 40% of the participants (N = 18) selected the introduction session. The main reasons provided for this were that participants indicated that they already had sufficient prior knowledge on the topic or that the topic was quite general in comparison to the other more specific training blocks. Twelve respondents selected the block Harmonisation in the framework of the VITALISE project. They evaluated this topic as less relevant to their own research and, consequently, not a priority in an already tight schedule. Respectively six and four researchers would not attend the methodology theoretical session or methodology workshop again. Provided reasons were the presence of prior knowledge on these topics or already having a clear idea about the protocol and methodologies that were suitable for their own research. Two participants indicated that a methodology workshop was not provided by their hosting living lab so they chose this option. Some participants indicated that they found it useful, but because they had to pick one they would opt for the methodology theory training. Finally, only three participants would skip the panel management session.

***Strengths of the training***

When asked what they appreciated most regarding the training, four categories of responses were identified. The exchange and interactions with the hosting living lab experts were mostly valued ( *N* = 21). Especially the open and interactive discussions, elicited by the presentations, made it possible to dig deeper into certain themes, methods, or struggles. Additionally, nineteen participants referred to specific content of the FTT training blocks. A wide variety of FTT topics was mentioned in this respect, which reflects the diversity of the interests of external researchers. The third category concerned the acquisition of new knowledge and insights in an efficient way ( *N* = 14). Finally, seven researchers mentioned the flexible, on demand approach of the FTT, with even the opportunity to have the training (partly) online.

Some additional strengths were mentioned in the general remarks section. Twenty-one participants were positive and thankful for the training and the opportunity to follow this training and to work with the VITALISE consortium. As one participant phrased it: “It was great to know the objectives of the living lab and how I can support them, and how they can support me.” Another participant declared that this initial training was necessary to be able to understand the VITALISE project as a whole as well as the specific living labs they were working with.

***Suggestions for additional topics***

Participants had the possibility to make suggestions for additional topics to include in the training. Several researchers indicated that they liked the program in the current state (N = 17), for example since it covered enough diverse topics already. Another thirteen participants responded that the question was not applicable or that they didn’t know. Suggestions that were provided by other participants were firstly to make the FTT even more interactive by adding not only small exercises but also more extensive workshops showcasing for instance the use of specific tools throughout the different training blocks. Topics could also be expanded with more descriptions of use cases conducted by the hosting living lab. Some specific topics that were deemed relevant to add, were (international) communication and collaboration, measuring impact, how researchers make use of living labs, to collaborate in research projects, how to write academic publications or reports, and technological as well as social innovations. Finally, finance and funding were also deemed important, as well as how to scale-up and commercialise projects or a section on cross-border data collection and data analysis.

Some additional suggestions were provided in the general remarks section. One participant wished for longer sessions with more in-depth discussions on their specific research project. Another participant suggested to add more case studies of the hosting living lab into the methodology section. This opinion was supported by the suggestion of another participant who had a preference for an even more interactive training. Some participants mentioned the need to gain insight into what kind of collaboration could be expected from Living Labs in future, referring to networking collaborations, opportunities to collaborate in future (EU-)projects or in shared value models.

***Evaluation of the NoteTheBook diary***

Participants were asked to rate their experience with the NoteTheBook diary on a scale from 0 (“not useful”) to 10 (“very useful”). Six participants did not provide a response to this question. Among the 43 participants who did respond, a substantial proportion ( *N* = 16) gave the maximum score of 10, indicating they found the diary very useful. Additionally, eight participants gave a high score of 8 or 9. Only three participants gave the diary a score lower than 5. Overall, participants were positive about the diary, with a mean score of 7.49 ( *SD* = 2.77).

## Discussion

Living Labs have demonstrated their potential for providing added value for innovation development ( Hossain *et al.*, 2019; Santonen *et al.*, 2024a; Ståhlbröst, 2013). However, many researchers and companies lack the knowledge to implement such methodologies in a sustainable way. This work presents and evaluates a novel Living Lab training program designed to onboard external researchers and familiarize them with Living Lab Research Infrastructures in the context of transnational visits and collaborations.

The current study introduces a flexible, short-term approach targeting more experienced audiences with existing expertise in research. This study proposes five fixed training blocks focused on introducing the participants to Living Labs, panel management, methodologies in theory and methodologies in practice and adding the element of harmonization that is considered important for research infrastructure users ( Santonen *et al.*, 2024b). The training was evaluated positively. Participants were very satisfied with the training and the quality and usefulness of all fixed blocks was rated high. After the training, most participants reported high confidence levels in applying Living Lab methodologies. The training was rated to be at least as they expected but mostly better than expected.

Moreover, experiential learning has demonstrated great potential in learning and training ( Hecht & Maass, 2008; Kang *et al.*, 2022). Many participants reported that the interactions with the trainers were a strength of the program. Suggestions for improvement mainly concerned making the training even more interactive, with workshops showcasing tools and instruments concerning all training themes. Some (more junior) researchers expressed the wish to have extra sessions with in-depth discussions on their own research project, whereas for other (senior) researchers this was less relevant.

Participants also differed in which specific training blocks they appreciated most, which illustrates that not all participants had the same needs in terms of training. These findings underscore the importance of offering flexible and tailored training programs that address both fundamental knowledge and practical application, ensuring relevance to the diverse needs of researchers in Living Lab environments. For example, while the introduction to Living Labs received the highest relevance scores before the training, it was also the block that most participants selected to remove when they had to. This appears to relate to the fact that this is the most general block and other blocks will extend insights from this block hereby potentially making it somewhat redundant when the complete training is provided. This ‘on demand’ choice of training blocks based on the researcher’s needs was very much appreciated. To enhance the training flexibility, the inclusion of one open block was also proposed that can be personalized to the user needs and adapted to the Living Lab expertise. As the need for assistance and the time required to process new information differ between novices and experts ( Persky & Robinson, 2017), the importance of tailoring the content and delivery of the program to learners’ levels of expertise is acknowledged. However, given that Living Labs have faced challenges in economic sustainability ( Fauth *et al.*, 2024; Paskaleva & Cooper, 2021), careful attention must be paid to the cost-effectiveness of the FTT program.

A significant challenge in the effective implementation of Living Labs lies in the lack of unified evaluation frameworks, which has been widely recognized in the literature ( Chen & Chou, 2010; Kovács, 2015). A set of harmonized criteria for Living Lab evaluation have been recently proposed and put into practice by ENoLL ( Vervoort *et al.*, 2022). Aligning the training aspects and modules with these criteria is crucial for the systematic advancement of Living Lab research. The FTT program presented in this study incorporates training components that emphasize not only the theoretical foundations but also practical aspects such as governance, participant engagement, and methodological adaptability; dimensions identified as critical for Living Lab evaluation ( Berberi *et al.*, 2023; Hossain *et al.*, 2019). The modular design of the training program aligns with recommendations to adapt evaluation models to diverse contexts and participant needs, ensuring relevance and applicability. Additionally, developing practical toolkits for Living Labs and their evaluation can provide valuable resources for establishing new Living Labs and projects, supporting wider uptake and standardized best practices ( Berberi *et al.*, 2023).

Some other recent initiatives have also started to address the gap in training, such as the international training school on “Knowledge transfer in and through Living Labs” which emphasized peer learning and methodological exchange across Living Lab practitioners ( Rohde *et al.*, 2024). Recently ENoLL has also launched the ENoLL Living Labbers Academy aiming at expanding the knowledge base of interested parties at a newcomer, intermediate, advanced, and expert level. Such a training offered by an international non-profit network organization can be an important step towards developing a systematic training approach for Living Lab methodologies that still remain scarce. A study by Diamantis *et al.*, 2024 has also proposed the design of a master course curriculum targeting innovation through Living Labs. In alignment with the proposed FTT structure, the curriculum included sections like panel management and the Living Lab methodology, including co-creation sessions, focus groups, experimentation and validation techniques. Konstantinidis *et al.* (2021) focused on the introduction of Living Lab concepts such as co-creation to undergraduate students with promising results from the evaluation from the students’ perspective, focusing again on co-creation aspects but adapting to a less experienced audience.

There are also some limitations to this study. The training could be adapted to the needs of the participants and was provided by local living lab staff. While all trainers had been informed about the training protocol and used the same training materials, there will have been differences in the exact operationalization of the training and blocks. Such a flexible tailor-made approach can be a strength for participants but also reduces standardization and can complicate training evaluation. The study used a small sample and it would beneficial to continue training initiatives and continuously evaluate and improve the content. The current training was offered free of charge in the context of subsidised collaborative research activities. This could have biased results. Finally, the survey responses reflected participants’ perceptions at a single time point, and thus, the long-term impact and usefulness of applying the knowledge in practice could not be measured. A follow-up study is recommended to assess sustained effects and to identify potential new Living Lab projects initiated by FTT program participants.

This study contributes to the growing body of work aimed at promoting user-centred approaches in research and innovation management. A flexible, modular training program was designed to help cater to the diverse needs of researchers. The positive evaluation results and high participant confidence levels underscore the added value of experiential and customizable learning formats. By aligning training content with emerging harmonization efforts and evaluation frameworks, such as those proposed by ENoLL, the program supports the systematic advancement of Living Lab methodologies. Continued development of structured, scalable, and context-sensitive training programs, supported by international collaborations and standardized approaches, will be essential for fostering sustainable and impactful Living Lab research across disciplines and borders.

## Ethics statement

The current procedures and materials were presented to the VITALISE internal ethical board and the data controller. Further ethical approval was not sought for the present study because it was an observational study collecting non-medical and non-sensitive data (feedback on training) from adult participants who voluntarily applied to take part in the VITALISE Horizon Europe project to participate in Transnational Access activities. All participants were informed about Transnational Access, the training, and the evaluation questionnaire in a service agreement which was signed by both the Horizon project lead and the visiting researcher. All participants provided informed consent in this service agreement. During data collection and analysis, the researchers complied to these guidelines from the internal ethical board and hereby to ethical standards from the Declaration of Helsinki and national and international guidelines for research.

## Data Availability

Repository: Accelerating Expertise: A Fast-Track Training Program for Living Lab Methodologies - supplementary materials. https://doi.org/10.17605/OSF.IO/N2DZB. Helsen *et al.* (2025) This project contains the following underlying data: Training evaluation dataset (csv file containing the responses from the participants on the training evaluation questionnaire) Data are available under the terms of the Creative Commons Zero "No rights reserved" data waiver (CC0 1.0 Public domain dedication). Repository: A Fast-Track Training Program for Living Lab Methodologies - supplementary materials. https://doi.org/10.17605/OSF.IO/N2DZB. Helsen *et al.* (2025) In accordance with the FAIR principles of open science, the processing of the results presented in Table 3 have been done through EOSC-RAISE platform, The Research Analysis Identifier (RAI) https://doi.org/21.T15999/raise/126 contains the results and the processing script applied on the dataset. This project contains the following extended data: Description_questions_and_response_options (word file describing the survey questions, response options, and variable names). Data are available under the terms of the Creative Commons Zero "No rights reserved" data waiver (CC0 1.0 Public domain dedication).
