# Supplementary material for: A Fast-Track Training Program for Living Lab Methodologies: program components, acceptability, and participant perceptions
Source: Open Res Eur. 2026 Jun 13;5:343. Originally published 2025 Nov 10. [Version 3] doi: 10.12688/openreseurope.21200.3 (PMC13129519; doi:10.12688/openreseurope.21200.3)
Supplement: Peer review history

# Reviewer report: Reviewer response for version 3

**DOI:** 10.21956/openreseurope.26221.r75846

**Published:** 2026-07-07

**Authors:** Sonia Massari

**Affiliations:** 1 University of Pisa, Pisa, Italy

I have carefully reviewed the revised version of the manuscript and the changes made by the authors in response to the previous comments and recommendations.

 I am satisfied with the improvements introduced. The authors have adequately addressed the main issues raised during the review process ( my requests), and the manuscript has been strengthened accordingly.

 I therefore recommend accepting the paper in its current form, with no further requests for revision.

If applicable, is the statistical analysis and its interpretation appropriate?

I cannot comment. A qualified statistician is required.

Is the study design appropriate and is the work technically sound?

Yes

Is the work clearly and accurately presented and does it engage with the current literature?

Yes

Are the conclusions drawn adequately supported by the results?

Yes

Are sufficient details of methods and analysis provided to allow replication by others?

Partly

Are all the source data and materials underlying the results available?

Partly

Reviewer Expertise:

NA

I confirm that I have read this submission and believe that I have an appropriate level of expertise to confirm that it is of an acceptable scientific standard.

# Reviewer report: Reviewer response for version 3

**DOI:** 10.21956/openreseurope.26221.r75851

**Published:** 2026-07-02

**Authors:** Phoebe Koundouri

**Affiliations:** 1 Athens University of Economics and Business, Athens, Greece

The authors have addressed my feedback. The article is ready for indexing.

If applicable, is the statistical analysis and its interpretation appropriate?

Not applicable

Is the study design appropriate and is the work technically sound?

Partly

Is the work clearly and accurately presented and does it engage with the current literature?

Yes

Are the conclusions drawn adequately supported by the results?

Partly

Are sufficient details of methods and analysis provided to allow replication by others?

Partly

Are all the source data and materials underlying the results available?

Yes

Reviewer Expertise:

NA

I confirm that I have read this submission and believe that I have an appropriate level of expertise to confirm that it is of an acceptable scientific standard.

# Reviewer report: Reviewer response for version 2

**DOI:** 10.21956/openreseurope.25592.r73762

**Published:** 2026-05-11

**Authors:** Stephen Gilbert, Anett Schönfelder

**Affiliations:** 1 TUD Dresden University of Technology, Dresden, Germany; 2 EKFZ, Technische Universitat Dresden Medizinische Fakultat Carl Gustav Carus, Dresden, Saxony, Germany

Dear editors and authors,

 Thank you for the opportunity to review your revised manuscript, which presents and evaluates the Fast-Track Training (FTT) programme developed within the VITALISE Horizon 2020 project, designed to onboard external researchers to Living Lab methodologies in the context of transnational access visits and collaborations. This is a timely and relevant contribution to a field that remains underserved in terms of structured, replicable training initiatives for researchers engaging with Living Lab infrastructures.

 While the authors clearly have made an effort to address the concerns raised during the first review round, a number of issues remain. I outline these below, organised by major and minor concerns.

**Major Comments:**

1. Length and focus of the introduction

The introduction is considerably lengthy and contains material that does not directly contribute to positioning this study. Several paragraphs elaborate on general Living Lab challenges and educational approaches at a level of detail that exceeds what is needed to motivate a training evaluation study. I would recommend reducing the introduction by approximately one third, retaining only content that directly leads to the rationale for the FTT program and the specific research questions.

 2. Participant numbers and training exposure 102 researchers were invited to complete the pre-survey, of whom 91 responded, and 49 subsequently completed the post-training evaluation. Do these 91 pre-survey respondents represent the total population of researchers who participated the FTT? Since the program was modular and tailored, not all participants received every training block. It would be useful to have a training exposure table with a total number of participants for each FTT block and information whether any block was entirely omitted at one or more sites. This would substantially improve the interpretability of the results.

 3. Inconsistency regarding online delivery Figure 1 states that "Participants receive training during transnational access", which implies exclusively in-person delivery. However, the training and data collection procedure section states that "the training was offered during the transnational access visit or online", and the strengths section refers to participants valuing "the opportunity to have the training (partly) online". The authors should engage with this discrepancy (e.g., revise Figure 1 to reflect that delivery could be in-person or online), and should clarify in the text which specific blocks or proportions of the training were delivered online versus on-site, and whether this varied systematically across hosting Living Labs.

 4. Discussion The discussion appropriately references a number of recent initiatives that address a similar gap in Living Lab training, including the international training school on “Knowledge transfer in and through Living Labs” and the ENoLL Living Labbers Academy. However, the manuscript does not engage analytically with how the FTT program differs from, improves upon, or complements these efforts. Additionally, although the limitations section has been meaningfully expanded throughout the review process, it does not substantively interrogate how the small and largely academic sample, the self-selection bias, the absence of a pre-training baseline, and the variability in program delivery constrain the scope and transferability of these conclusions.

**Minor Comments:**

1. Missing information about participant background

Table 2 reports participants' professional domain and the text reports gender, but no information is provided on career level (e.g., PhD student, postdoctoral researcher), educational degree, or age. This is particularly relevant given that the authors themselves note that trainee profile influences learning outcomes, and to assess whether the heterogeneity of the sample may have influenced the results.

 2. Insufficient description of the free block The free block is described as content determined by the hosting Living Lab's own expertise or best practices, with only a single example provided (a training block on working with companies and business model preparation, as delivered in Belgium (a Living Lab for which only one FTT evaluation survey was completed, with the total number of participants being unclear, as highlighted in point 2)). Given that this block was offered across several Living Lab sites, the reader has no way to understand what the range of content actually looked like across the program. I therefore encourage to add a brief summary or table of the free block topics delivered at each hosting Living Lab.

 3. Capitalisation of “Living Lab” Throughout the manuscript, capitalisation of the term "Living Lab" remains inconsistent. In some instances, it is capitalised (e.g., "Living Lab methodology," "Living Lab infrastructure"), while in others it appears in lowercase (e.g., "living lab staff," "hosting living lab," "living lab research"). Given that "Living Lab" functions as a proper noun and established concept within this community, consistent capitalisation should be applied throughout the manuscript.

If applicable, is the statistical analysis and its interpretation appropriate?

Partly

Is the study design appropriate and is the work technically sound?

Partly

Is the work clearly and accurately presented and does it engage with the current literature?

Partly

Are the conclusions drawn adequately supported by the results?

Partly

Are sufficient details of methods and analysis provided to allow replication by others?

Partly

Are all the source data and materials underlying the results available?

Yes

Reviewer Expertise:

Living Labs in the Health domain

We confirm that we have read this submission and believe that we have an appropriate level of expertise to confirm that it is of an acceptable scientific standard, however we have significant reservations, as outlined above.

# Response

**Published:** 2026-06-02

**Authors:** Nele De Witte

**Affiliations:** Centre of Expertise Care and Well-being, Thomas More University of Applied Sciences, Geel, Belgium

Dear Prof. Gilbert, Dear Ms. Schönfelder,

 Thank you for your thorough reading of the manuscript and allowing us to further clarify certain elements of the study. We have tried to limit the extent of additional changes to the manuscript after approval of other reviewers, but have published a new version of the manuscript and have carefully addressed all concerns in this reviewer response.

**Reviewer: **Length and focus of the introduction The introduction is considerably lengthy and contains material that does not directly contribute to positioning this study. Several paragraphs elaborate on general Living Lab challenges and educational approaches at a level of detail that exceeds what is needed to motivate a training evaluation study. I would recommend reducing the introduction by approximately one third, retaining only content that directly leads to the rationale for the FTT program and the specific research questions.

***Response: **Since the other 5 reviewer did not advise for abbreviating the introduction, we have decided to not abbreviate it at this time (to avoid removing sections that were informative for other researchers). The length does not seem excessive and we prefer not too many too many substantial changes at this stage.*

**Reviewer:** Participant numbers and training exposure 102 researchers were invited to complete the pre-survey, of whom 91 responded, and 49 subsequently completed the post-training evaluation. Do these 91 pre-survey respondents represent the total population of researchers who participated the FTT? Since the program was modular and tailored, not all participants received every training block. It would be useful to have a training exposure table with a total number of participants for each FTT block and information whether any block was entirely omitted at one or more sites. This would substantially improve the interpretability of the results.

***Response:** All individuals who were selected for transnational access were invited to participate but not all completed the survey. Adding such an exposure table was also suggested by reviewer 1 but, it is no possible to provide this. As mentioned in our response to reviewer 1: No rigorous implementation documentation framework was adopted to document training exposure so we do not have exact statistics on program content presented to each participant. We acknowledge that this is a limitation: “ However, while variation in implementation limited precise assessment of exposure, it reflects real-world practice, and positive perceptions across sites suggest the program’s acceptability across diverse conditions.”*

**Reviewer:** 3. Inconsistency regarding online delivery Figure 1 states that "Participants receive training during transnational access", which implies exclusively in-person delivery. However, the training and data collection procedure section states that "the training was offered during the transnational access visit or online", and the strengths section refers to participants valuing "the opportunity to have the training (partly) online". The authors should engage with this discrepancy (e.g., revise Figure 1 to reflect that delivery could be in-person or online), and should clarify in the text which specific blocks or proportions of the training were delivered online versus on-site, and whether this varied systematically across hosting Living Labs. ***Response: **We have now updated figure 1 to clarify that delivery was either in-person or online.*

**Reviewer:** 4. Discussion The discussion appropriately references a number of recent initiatives that address a similar gap in Living Lab training, including the international training school on “Knowledge transfer in and through Living Labs” and the ENoLL Living Labbers Academy. However, the manuscript does not engage analytically with how the FTT program differs from, improves upon, or complements these efforts. Additionally, although the limitations section has been meaningfully expanded throughout the review process, it does not substantively interrogate how the small and largely academic sample, the self-selection bias, the absence of a pre-training baseline, and the variability in program delivery constrain the scope and transferability of these conclusions.

***Response: **The program was designed to operate within the context of the Vitalise pogram, it was co-created with project partners to fit the Transnational Access program. It was not designed to replace or compete with existing programs (which do not always have their content published openly, making it hard to compare approaches exactly). By making our materials and processes entirely open, comparisons can be made with future programs and materials can be used by others. In the limitations section we indeed acknowledge challenges relating to generalization and mention elements such as self-selection bias and limitations of the design and implementation. Since it is not possible to assess the extent to which such factors actually changed results and there is no one solution to solving this in future research, we hope that readers can use the current data, interpretations, and future perspectives to build new and improved versions of such training programs in the future.*

**Minor Comments:** **Reviewer:** Missing information about participant background Table 2 reports participants' professional domain and the text reports gender, but no information is provided on career level (e.g., PhD student, postdoctoral researcher), educational degree, or age. This is particularly relevant given that the authors themselves note that trainee profile influences learning outcomes, and to assess whether the heterogeneity of the sample may have influenced the results.

***Response: **We indeed noted in the introduction that trainee profile can influence learning. This was the reason why we decided to allow for flexibility in delivery of the training program. We did not collect data on participants’ academic or practice experience. Given the small sample size, sample heterogeneity, and flexibility in training implementation, subgroup analyses were not foreseen (or advised) and the study is accordingly framed around acceptability and perceptions.*

**Reviewer:** 2. Insufficient description of the free block The free block is described as content determined by the hosting Living Lab's own expertise or best practices, with only a single example provided (a training block on working with companies and business model preparation, as delivered in Belgium (a Living Lab for which only one FTT evaluation survey was completed, with the total number of participants being unclear, as highlighted in point 2)). Given that this block was offered across several Living Lab sites, the reader has no way to understand what the range of content actually looked like across the program. I therefore encourage to add a brief summary or table of the free block topics delivered at each hosting Living Lab.

***Response: **Thank you for your suggestions. As mentioned in the limitations, we do not have exact statistics on program content presented to each participant and we, therefore, cannot deliver an exhaustive list of exposure  to block topics. We, therefore, decided to limit ourselves to providing an example for a block that was included in the available open access training materials.*

**Reviewer:** 3. Capitalisation of “Living Lab” Throughout the manuscript, capitalisation of the term "Living Lab" remains inconsistent. In some instances, it is capitalised (e.g., "Living Lab methodology," "Living Lab infrastructure"), while in others it appears in lowercase (e.g., "living lab staff," "hosting living lab," "living lab research"). Given that "Living Lab" functions as a proper noun and established concept within this community, consistent capitalisation should be applied throughout the manuscript.

***Response: **We have now consistently capitalized the word in the manuscript. Thank you for your suggestions and we hope we have been able to answer your questions in a satisfactory way.*

# Reviewer report: Reviewer response for version 2

**DOI:** 10.21956/openreseurope.25592.r72970

**Published:** 2026-05-06

**Authors:** Juan Pablo Centeno

**Affiliations:** 1 KU Leuven, Public Governance Institute (PGI), Leuven, Belgium

Dear editors and authors, thank you for the invitation to review this paper. I have read it with great interest and I believe it is a key contribution to capacity-building process in the implementation and operation of Living Labs.

 The paper presents an assessment of a training programme aimed at Living Lab researchers and practitioners in order to build capacities in key aspects of Living Labs, such as user-centred research, living lab practices and co-design. This is a relevant topic, particularly in the context of a fats growing Living Lab community in Europe.

 As such, the paper responds to both practical and research-related issues: on the one hand, there is a need to further build capacities and skills among individuals and organisations in order to implement successful Living Lab initiatives. On the other hand, there is a growing body of literature on Living Labs suggesting that skills such as facilitation, co-creation, panel management and user-engagement are key factors shaping the success of Living Labs. The paper explicitly and clearly engages with this scholarship, while acknowledging the many challenges faced by Living Labs face and by researchers/managers/practitioners, according to literature.

 The study builds empirically on self-reported data from the assessment of a fast-track training programme on Living Labs. This comprises several rounds of survey (pre and post training), mid-term evaluation, among other instruments that provide qualitative and quantitative data. It covers a broad range of Living Lab organisations and participants, which enhances the potential scalability of results.

 Further reflection on potential generalisability and scalability of results could further enhance the discussion of the paper. While the authors acknowledge that “To ensure generalizability, It would be beneficial to continue training initiatives and continuously evaluate and improve the content, especially when transferring to different contexts”, further detail could be provided on ‘how’ these training initiatives should be continued and evaluated in order to consider diversity of organisational, country, and sectoral contexts.

 The procedures and methods are clearly detailed in the methods section. Further reflection on response rates could guide the reader in interpreting the scope of the results.

 Finally, the discussion and conclusions section provide comprehensive reflection based on the results from the surveys. I recommend further engaging with the implications related to the aspects and themes identified by participants as lacking from the training. I wonder how such topics (e.g. “communication and collaboration, measuring impact, how researchers make use of living labs, to collaborate in research projects, how to write academic publications or reports, and technological as well as social innovations”) relate to current challenges in the implementation and management of Living Labs. The paper could provide additional reflection on this matter, as well as on the strengths identified by participants in the programme and how such strengths reflect key success factors identified by literature in relation to Living Labs.

If applicable, is the statistical analysis and its interpretation appropriate?

Yes

Is the study design appropriate and is the work technically sound?

Yes

Is the work clearly and accurately presented and does it engage with the current literature?

Yes

Are the conclusions drawn adequately supported by the results?

Yes

Are sufficient details of methods and analysis provided to allow replication by others?

Yes

Are all the source data and materials underlying the results available?

No source data required

Reviewer Expertise:

Living Labs; knowledge management; collaborative governance

I confirm that I have read this submission and believe that I have an appropriate level of expertise to confirm that it is of an acceptable scientific standard.

# Response

**Published:** 2026-06-01

**Authors:** Nele De Witte

**Affiliations:** Centre of Expertise Care and Well-being, Thomas More University of Applied Sciences, Geel, Belgium

Dear Mr. Centeno,

 Thank you for reading the revised manuscript and providing your reflections. To further clarify the further question you had, the training was conducted in the context of Vitalise, a Horizon consortium included the European network of Living Labs (ENoLL). They will be able to use the current insights into their training programs and are positioned well to evaluate such training initiatives. However, we would like to invite everybody with an interest in this field to use the materials do provide further training to the field. This also includes the feedback we received. For new implementations, training priorities can be determined based on the feedback of the current participants, including both the strengths and the missing themes. Since the Vitalise project is now finished, we are not able to implement a new version of the training materials taking into account the feedback from the participants, but we hope that others can build on the work to help implement living lab training programs and link training outcomes to living lab success factors.

# Reviewer report: Reviewer response for version 2

**DOI:** 10.21956/openreseurope.25592.r72806

**Published:** 2026-04-29

**Authors:** Phoebe Koundouri

**Affiliations:** 1 Athens University of Economics and Business, Athens, Greece

The paper is ready to be indexing now, after the indicated changes.

If applicable, is the statistical analysis and its interpretation appropriate?

Not applicable

Is the study design appropriate and is the work technically sound?

Partly

Is the work clearly and accurately presented and does it engage with the current literature?

Yes

Are the conclusions drawn adequately supported by the results?

Partly

Are sufficient details of methods and analysis provided to allow replication by others?

Partly

Are all the source data and materials underlying the results available?

Yes

Reviewer Expertise:

NA

I confirm that I have read this submission and believe that I have an appropriate level of expertise to confirm that it is of an acceptable scientific standard.

# Reviewer report: Reviewer response for version 1

**DOI:** 10.21956/openreseurope.22933.r65183

**Published:** 2026-01-20

**Authors:** Francesca Torlone

**Affiliations:** 1 Universita degli Studi di Firenze, Florence, Tuscany, Italy

The manuscript presents the design, implementation, and evaluation of a fast-track training programme developed within the Horizon 2020 VITALISE project, aimed at enhancing researchers’ competencies in Living Lab methodologies. The topic is relevant and timely, particularly in the context of European research infrastructures, user-centred innovation, and Responsible Research and Innovation (RRI).

 However, while the paper is clearly written and well documented, its contribution remains largely *descriptive.*

 The manuscript addresses a genuine gap in the Living Lab ecosystem, namely the lack of structured training initiatives for researchers accessing Living Lab infrastructures that may improve the link between research, innovation, labour, civil society. This is an important issue and one that the paper stresses.

 Nonetheless, the original scientific contribution of the paper is currently limited. The manuscript mainly documents (i) the structure of the training programme, (ii) participants satisfaction, (iii) and self-reported confidence outcomes. What is missing is a clearer articulation of what new analytical insights this study offers to the existing literature on Living Labs, co-creation, and research capacity building. 

 A major limitation of the paper is its predominantly descriptive nature. While the authors report extensive quantitative and qualitative feedback from participants, the analysis remains largely at the level of satisfaction scores and thematic summaries.

 In particular, the paper does not sufficiently analyse *why* the training appears to work, nor does it explore and analyse the learning processes that are being developed (and related positive and negative learning outcomes), ****or skill acquisition beyond participants’ perceptions. The reliance on self-reported confidence measures, without triangulation or longitudinal follow-up, further limits the analytical strength of the conclusions.As a result, the findings confirm that participants appreciated the programme, but they do not convincingly demonstrate how or under which conditions the training contributes to sustained methodological competence and to ameliorate the cooperation and capacity building of actors involved.

 A key conceptual issue concerns the use of the term **“** *fast-track training programme* **”**.

 In the broader international literature, *fast-track training* and *fast-track integration* are widely used concepts, particularly in relation to education and training programmes addressed to asylum seekers, refugees, migrants for their fast integration into the labour market. In this sense, fast track integration implies deep theoretical and epistemological considerations affecting the learning quality of professional settings these targets are involved in. 

 The unqualified use of the term “fast-track” risks being conceptually misleading for readers and creates ambiguity regarding the intended meaning and scope of the concept. It might be considered to announce to this terminology to make clear the field of investigation. 

 The methodological approach is generally appropriate for an exploratory evaluation. Nevertheless, some aspects deserve more critical reflection: (i) Sample size and composition: The evaluation is based on a relatively small and predominantly academic sample, which limits the generalisability of the findings. (ii) Variability in programme delivery: While flexibility is presented as a strength, differences in how the training was delivered across Living Labs reduce standardisation and complicate comparative evaluation. (iii) Short-term perspective: The absence of follow-up data makes it impossible to assess longer-term impacts, such as actual application of Living Lab methodologies or the initiation of new collaborations.These limitations are partially acknowledged but should be more explicitly discussed in relation to the claims made in the conclusion.

 The manuscript is suitable for indexing provided that the authors: (i) Strengthen the analytical framing of the study; (ii) Clarify the conceptual use of “fast-track” in relation to existing literature; (iii) More critically discuss methodological limitations and their implications for the conclusions

If applicable, is the statistical analysis and its interpretation appropriate?

Partly

Is the study design appropriate and is the work technically sound?

Yes

Is the work clearly and accurately presented and does it engage with the current literature?

Partly

Are the conclusions drawn adequately supported by the results?

Partly

Are sufficient details of methods and analysis provided to allow replication by others?

Partly

Are all the source data and materials underlying the results available?

Yes

Reviewer Expertise:

My areas of research are adult education public policies, fast-track policies and practices in the labour market, adult education in professional settings, higher education systems and adult education, skills framework development

I confirm that I have read this submission and believe that I have an appropriate level of expertise to confirm that it is of an acceptable scientific standard, however I have significant reservations, as outlined above.

# Response

**Published:** 2026-04-08

**Authors:** Nele De Witte

**Affiliations:** Centre of Expertise Care and Well-being, Thomas More University of Applied Sciences, Geel, Belgium

Dear Prof. Torlone, Thank you very much for your thorough reading of the manuscript and your suggestions. We have made the necessary changes to the manuscript and have provided a point-by-reply below.  

**Reviewer:** The manuscript presents the design, implementation, and evaluation of a fast-track training programme developed within the Horizon 2020 VITALISE project, aimed at enhancing researchers’ competencies in Living Lab methodologies. The topic is relevant and timely, particularly in the context of European research infrastructures, user-centred innovation, and Responsible Research and Innovation (RRI).

 However, while the paper is clearly written and well documented, its contribution remains largely *descriptive.*

 The manuscript addresses a genuine gap in the Living Lab ecosystem, namely the lack of structured training initiatives for researchers accessing Living Lab infrastructures that may improve the link between research, innovation, labour, civil society. This is an important issue and one that the paper stresses.

 Nonetheless, the original scientific contribution of the paper is currently limited. The manuscript mainly documents (i) the structure of the training programme, (ii) participants satisfaction, (iii) and self-reported confidence outcomes. What is missing is a clearer articulation of what new analytical insights this study offers to the existing literature on Living Labs, co-creation, and research capacity building. 

**Response:** Thank you very much for your thorough reading and indication of relevance. We will address the suggestions below.

**Reviewer:** A major limitation of the paper is its predominantly descriptive nature. While the authors report extensive quantitative and qualitative feedback from participants, the analysis remains largely at the level of satisfaction scores and thematic summaries.

**Response: **As you mentioned above, not that many training programs are available in the field of living lab methodology. Therefore, the current paper aimed to present a training approach and its content which is flexible and accessible. A main goal of this contribution was therefore to describe a the content and development of a training . The second purpose of the study was to present self-reported experiences with the program. We have now updated the study aims in the introduction and the manuscript title to better reflect this.  

**Reviewer: **In particular, the paper does not sufficiently analyse *why* the training appears to work, nor does it explore and analyse the learning processes that are being developed (and related positive and negative learning outcomes), ****or skill acquisition beyond participants’ perceptions. The reliance on self-reported confidence measures, without triangulation or longitudinal follow-up, further limits the analytical strength of the conclusions.As a result, the findings confirm that participants appreciated the programme, but they do not convincingly demonstrate how or under which conditions the training contributes to sustained methodological competence and to ameliorate the cooperation and capacity building of actors involved.

**Response: **Based on the analyses that were performed, it is indeed not possible inform on gains in sustained methodological competence and underlying mechanisms of such gains. In the introduction, we have now provided more background on the concept of experiential learning and aspects that influence learning outcomes, e.g., trainee profile. While the training was built on these principles, it was designed to allow for flexible adoption (in terms of content and target population), which limits opportunities for formal evaluation. We have, therefore, added to the limitations that: “The study did not employ a formal pre–post design and relied on self-reported post-training perceptions, limiting conclusions about learning gains. Consequently, the findings should be interpreted as evidence of perceived usefulness, acceptability, and feasibility rather than validation of accelerating expertise or increasing confidence.”  

**Reviewer: **A key conceptual issue concerns the use of the term **“** *fast-track training programme* **”**.

 In the broader international literature, *fast-track training* and *fast-track integration* are widely used concepts, particularly in relation to education and training programmes addressed to asylum seekers, refugees, migrants for their fast integration into the labour market. In this sense, fast track integration implies deep theoretical and epistemological considerations affecting the learning quality of professional settings these targets are involved in. 

 The unqualified use of the term “fast-track” risks being conceptually misleading for readers and creates ambiguity regarding the intended meaning and scope of the concept. It might be considered to announce to this terminology to make clear the field of investigation. **Response: ** Thank you for this remark. This name was chosen to contrast this intense focused program with other extended educational approaches (e.g., the ENoLL virtual learning lab). We understand that this term has been used for other purposes as well and that this could cause some confusion in some readers. However, the term was consistently used in the Vitalise project and in the available document and believe therefore that changing it in this publication will only cause more confusion.  We have now added to the methods that “The term fast-track was used to indicate that it was a short, intense, and focused program.”.

**Reviewer: **The methodological approach is generally appropriate for an exploratory evaluation. Nevertheless, some aspects deserve more critical reflection: (i) Sample size and composition: The evaluation is based on a relatively small and predominantly academic sample, which limits the generalisability of the findings. (ii) Variability in programme delivery: While flexibility is presented as a strength, differences in how the training was delivered across Living Labs reduce standardisation and complicate comparative evaluation. (iii) Short-term perspective: The absence of follow-up data makes it impossible to assess longer-term impacts, such as actual application of Living Lab methodologies or the initiation of new collaborations. These limitations are partially acknowledged but should be more explicitly discussed in relation to the claims made in the conclusion.

**Response: **Thank you for these suggestions. We have expanded the limitations section, and it now includes more details on the sample and its limitations, implementation variations, and long-term impact. However, we acknowledge that these critical reflections should also be included in the conclusions, and this is now the case in the discussion (“However, further research is needed to establish formal learning gains and long-term impacts of the training, as well as explore learning gains in diverse samples.”) as well as the abstract (“However, further formal evaluation of learning gains is needed.”)

**Reviewer: **The manuscript is suitable for indexing provided that the authors: (i) Strengthen the analytical framing of the study; (ii) Clarify the conceptual use of “fast-track” in relation to existing literature; (iii) More critically discuss methodological limitations and their implications for the conclusions

**Response:** Thank you for your careful reading of the manuscript. We have improved the presentation of the study and discussion of the findings to make it clearer what the goals and limitations of the approach were. We have also generally improved the writing to guarantee that it is clear that we are discussing self-report outcomes. We believe that the manuscript has substantially improved and could help to inform the field about training content and experiences and hope it can inspire other researchers to develop and publish on their training initiatives.

# Reviewer report: Reviewer response for version 1

**DOI:** 10.21956/openreseurope.22933.r64101

**Published:** 2026-01-10

**Authors:** Phoebe Koundouri

**Affiliations:** 1 Athens University of Economics and Business, Athens, Greece

Training on LL methodologies and relevant capacity building are not sufficiently addressed until now. Thus, the paper deals with a very timely topic. The study has been part of a H2020 project, in this respect design and evaluation are appropriate.

 Following aspects require, however, attention:

1. The manuscript is rather descriptive. It provides information about the various aspects (the questionnaires, the modular training approach, the analysis of replies). But it lacks a more in depth methodological and theoretical analysis in relation to the challenges identified and why training on user-centred approaches can help overcoming them. This is even more important, as the manuscript implies that there are major differences between the various disciplines (healthcare vs a number of others).
2. The study is based on a very small sample, and it seems that only limited follow-up and evaluation activities took place. Again, sample and methodology are appropriate for H2020 project of this type, but it makes it difficult to claim that “This study shows that well-designed training programs can help researchers use Living Labs more successful, which could lead to better innovations that are more connected to society’s needs.”, as a general statement as this one would require more evidence, both in terms of participants and time.
3. Similarly to the point above, drawing broader conclusions (such as the statement in the Conclusion paragraph) would require not only a more systemic approach but also the provision of baseline figures and benchmarks. Moreover, if the study focussed only on the healthcare domain, how can we ensure that the outcomes are replicable and generalisable? This requires justification.
4. The positive replies in relation to increased confident by participants in applying Living Lab methodologies post-training, do not necessarily correspond to built capacity. The analysis should differentiate between self-claimed confidence and actual knowledge and capacity gained.
5. More information should be provided on how the participants have been recruited. The eligibility and exclusion criteria are clearly mentioned and are appropriate, but there is no information on demographics or on any previous LL knowledge and experience of the participants.

It is not clear what has been the added value in the whole process and the contribution by using a self-reflection diary. It seems that the participants have been asked to evaluate the Note: The Book diary from an end-user perspective (evaluate the application). But this doesn’t’ necessarily say something about the knowledge acquiring process.

If applicable, is the statistical analysis and its interpretation appropriate?

Not applicable

Is the study design appropriate and is the work technically sound?

Partly

Is the work clearly and accurately presented and does it engage with the current literature?

Yes

Are the conclusions drawn adequately supported by the results?

Partly

Are sufficient details of methods and analysis provided to allow replication by others?

Partly

Are all the source data and materials underlying the results available?

Yes

Reviewer Expertise:

NA

I confirm that I have read this submission and believe that I have an appropriate level of expertise to confirm that it is of an acceptable scientific standard, however I have significant reservations, as outlined above.

# Response

**Published:** 2026-04-08

**Authors:** Nele De Witte

**Affiliations:** Centre of Expertise Care and Well-being, Thomas More University of Applied Sciences, Geel, Belgium

Dear Prof. Koundouri, Thank you very much for your thorough reading of the manuscript and your suggestions. We have made the necessary changes to the manuscript and have provided a point-by-reply below.  

**Reviewer: **Training on LL methodologies and relevant capacity building are not sufficiently addressed until now. Thus, the paper deals with a very timely topic. The study has been part of a H2020 project, in this respect design and evaluation are appropriate.

 Following aspects require, however, attention:

 The manuscript is rather descriptive. It provides information about the various aspects (the questionnaires, the modular training approach, the analysis of replies). But it lacks a more in depth methodological and theoretical analysis in relation to the challenges identified and why training on user-centred approaches can help overcoming them. This is even more important, as the manuscript implies that there are major differences between the various disciplines (healthcare vs a number of others).

**Response: **Thank you for your invitation to elaborate more on the challenges related to training on living methodologies. We have now extended the introduction to better frame the need for training, especially in the healthcare field.  

**Reviewer:**

 The study is based on a very small sample, and it seems that only limited follow-up and evaluation activities took place. Again, sample and methodology are appropriate for H2020 project of this type, but it makes it difficult to claim that “This study shows that well-designed training programs can help researchers use Living Labs more successful, which could lead to better innovations that are more connected to society’s needs.”, as a general statement as this one would require more evidence, both in terms of participants and time.

**Response: **We have now adjusted this claim in the Plain Language summary to more accurately represent study outcomes: “This study suggests that researchers find a carefully designed and modular training programs useful to provide more insight into Living Lab methodologies.” The title has also been changed, and the limitations were expanded so as to not to overstate results.  

**Reviewer:**

 Similarly to the point above, drawing broader conclusions (such as the statement in the Conclusion paragraph) would require not only a more systemic approach but also the provision of baseline figures and benchmarks. Moreover, if the study focussed only on the healthcare domain, how can we ensure that the outcomes are replicable and generalisable? This requires justification.

**Response: **We have adjusted the language in the manuscript to better reflect the nature of our findings. We have also added to the limitations that: “The study used a small sample (not including all training participants which implies vulnerability for self-selection) and was situated within the healthcare domain. It would be beneficial to continue training initiatives and continuously evaluate and improve the content, especially when transferring to different contexts.”. In the newly added section on economic sustainability, we have also briefly indicated that some time might be needed to familiarize oneself with the content and reflect on experience within the trainer’s own field.  

**Reviewer:**

 The positive replies in relation to increased confident by participants in applying Living Lab methodologies post-training, do not necessarily correspond to built capacity. The analysis should differentiate between self-claimed confidence and actual knowledge and capacity gained.

**Response: **We have now adjusted our wording to make it clearer that the results are self-reported outcomes and that we did not measure actual knowledge and capacity gained. The limitations section now also reads: “The current study did not use formal assessment of learning gains but relied on a self-report post-training survey to evaluate the training.”  

**Reviewer:**

 More information should be provided on how the participants have been recruited. The eligibility and exclusion criteria are clearly mentioned and are appropriate, but there is no information on demographics or on any previous LL knowledge and experience of the participants.

**Response: **Unfortunately, we did not collect data on previous LL knowledge and experience. We did not include all demographics for the participants in the expectations survey (since the main focus of the manuscript concerns the FTT evaluation. We have, however, now assessed and reported that the full sample of invited researchers matches the FTT evaluation participants in gender and background.

**Reviewer: **It is not clear what has been the added value in the whole process and the contribution by using a self-reflection diary. It seems that the participants have been asked to evaluate the Note: The Book diary from an end-user perspective (evaluate the application). But this doesn’t’ necessarily say something about the knowledge acquiring process. **Response: **Previous research has suggested that a self-reflection diary can be an effective technique for learning. However, in the current study NoteTheBook was an optional tool which was provided to participants. Since we did not monitor use, we disclosed it here for transparency rather than to provide elaborate insights into its use and effectiveness. We have now removed some details and included in the general usefulness section of the results as opposed to a separate section . Thank you for these suggestions, we believe that the integration of your feedback has significantly improved the manuscript.

# Reviewer report: Reviewer response for version 1

**DOI:** 10.21956/openreseurope.22933.r65564

**Published:** 2026-01-10

**Authors:** Sonia Massari

**Affiliations:** 1 University of Pisa, Pisa, Italy

The manuscript presents and evaluates a modular training programme, defined as *fast-track* due to its limited duration, developed within the H2020 VITALISE project to support external researchers in accessing transnational Living Lab (LL) infrastructures in the *Health & Wellbeing* domain.

 The contribution is practical, well contextualised, and provides useful documentation of a capacity-building initiative aimed at methodological harmonisation and adoption across Living Labs. However, the evaluation framework is largely perceptual in nature (satisfaction, perceived usefulness, post-training self-efficacy) and does not fully support some of the implicit inferences present in the framing of the paper (e.g. “accelerating expertise”, “increased confidence”), which should be more carefully aligned with the limits of the available evidence.

 At the same time, it is important to underline that the paper addresses a relevant and underexplored gap in the literature, namely the training and capacity building of professionals—particularly researchers—working within Living Lab contexts. From this perspective, the manuscript represents an interesting milestone in the emerging body of literature concerned with methodologies and methods in Living Lab research and practice.

 The paper effectively highlights a real and well-recognised issue: the gap between the increasing availability of Living Lab infrastructures and the competencies required to use them in a robust, reflective, and sustainable manner. The conceptual framing that links Living Labs, co-creation, Responsible Research and Innovation (RRI), and the need for dedicated training is convincing and well grounded.

 The structure of the programme is presented through a clear sequential logic (Figure 1) and a detailed description of the training blocks (Figure 2), including welcoming and icebreaker activities, an introduction to Living Labs and ethics, VITALISE and harmonisation, methodology (theory), a methodology workshop, panel management, and an optional open block. The modular and adaptable nature of the programme represents a clear strength, particularly in relation to heterogeneous contexts and hosting Living Labs.

 The open orientation of the initiative is also commendable. The availability of public training materials, datasets, and extended resources enhances the replicability and transferability of the programme.

 Quantitative results report very high levels of satisfaction (mean 9.11/10), high perceived quality and usefulness across training blocks (approximately 4.2–4.8/5), and high post-training confidence (8.54/10). Qualitative feedback further emphasises strengths such as interaction with experts, learning efficiency, and flexibility of the format.

 Critical issues and recommended improvements:

 The manuscript is supported by a rich and generally well-organised body of literature that provides a clear introduction to Living Labs, wicked problems, and the adaptability of the Living Lab model to contemporary societal challenges characterised by situated, real-life, and multi-actor conditions. This framing effectively prepares the reader for the proposed intervention.

 However, I would expect a more substantial engagement with the scientific literature on competencies and capacity building, particularly with regard to the evolution of roles, skills, and professional profiles required within Living Labs. This discussion could usefully address capacity building from the perspective of the different actors involved (e.g. researchers, facilitators, managers, institutional partners), rather than remaining primarily implicit.

 The title and several passages in the manuscript suggest an effect in terms of *“accelerating expertise”* and/or increasing confidence or competence. However, the evaluation relies primarily on:

- satisfaction measures,
- perceived quality and usefulness,
- post-training confidence only (without a pre-training baseline),
- open-ended qualitative feedback.

In its current form, the evaluation design allows the authors to state that the training is well accepted, perceived as useful, and associated with high self-reported confidence after completion. It does not, however, support causal claims regarding an *increase* in competence, nor does it justify references to *expertise* in a strong sense (i.e. skill acquisition or demonstrable capability development).

 For this reason, a systematic revision of the language used across the title, abstract, and discussion is recommended. Alternative formulations such as *onboarding effectiveness*, *acceptability*, *perceived readiness*, *perceived competence*, or *self-reported post-training confidence* would more accurately reflect what is empirically measured.

 The programme is explicitly designed to be adaptable: not all participants receive all training blocks, and some modules may be abbreviated or delivered online. While this flexibility is a clear operational strength, it introduces methodological challenges that are not sufficiently addressed in the current reporting.

 Key details that are currently missing include:

- which training blocks were delivered to which participants or sites,
- actual duration of each block in practice,
- delivery mode (in-person vs online),
- number of responses per block and overlap among respondents.

To improve interpretability, I strongly recommend the inclusion of a summary table (for instance organised by site or participant profile) detailing training exposure, delivery mode, approximate duration, and corresponding sample sizes.

 There is a substantial difference between the number of respondents to the expectations survey (N = 91) and the post-training evaluation (N = 49). It is both plausible and methodologically relevant that more satisfied participants are more likely to complete the evaluation (self-selection), and that the free-of-charge nature of transnational access may further amplify positive responses.

 While the manuscript partially acknowledges this issue, it would benefit from a more explicit treatment. I suggest including a participant flow diagram or structured graphical presentation of the participation pathway (invited → pre-survey respondents → training participants → post-survey respondents). Where possible, a minimal comparison between respondents and non-respondents (e.g. by domain, site, country, or seniority) would further strengthen transparency and allow readers to better assess potential biases.

 At a narrative level, the manuscript appears to oscillate between two objectives:

1. describing a replicable modular training model, and
2. demonstrating its effectiveness in building expertise.

The second objective is not fully supported by the current evaluation design. I therefore recommend making explicit that the primary contribution lies in the design and acceptability / readiness evaluation of a multi-site modular training programme, rather than in demonstrating learning outcomes or expertise development.

 Relatedly, the description of the training model remains partial. Beyond directing readers to online tools, the paper does not clearly articulate explicit learning outcomes for each module. Making these outcomes explicit would significantly enhance transferability and would also enable future, more objective forms of evaluation (e.g. rubrics, protocol assessment, or other forms of structured assessment).

 In addition, the diary component is conceptually interesting but remains marginal in the current manuscript. Its evaluation focuses on perceived usefulness, without reporting even minimal indicators of actual adoption (e.g. frequency of use, extent of annotation). The authors should therefore make a clearer choice: either integrate the diary as a core component, supported by appropriate usage indicators, or reposition it as an additional element within the broader training toolkit.

 As a final, non-essential but valuable improvement, the paper rightly raises the issue of the economic sustainability of Living Labs, yet provides no operational indicators related to the training itself (e.g. trainer hours, preparation effort, indicative costs). Even minimal data in this respect would lend greater credibility to the discussion of scalability and cost-effectiveness.

 Overall, the manuscript represents a useful and publishable contribution as an *intervention description combined with an acceptability evaluation*, situated within a real transnational context. It demonstrates a good level of transparency and is supported by a convincing modular training structure. The work clearly offers added value to the field.

 However, for the manuscript to be fully suitable for publication, a number of targeted revisions are required. In particular, these concern a clearer realignment between claims and evidence, greater detail regarding variability in programme delivery, and a more explicit handling of selected reporting aspects, as outlined above.

 With these improvements, the paper would constitute a solid and methodologically sound contribution to the literature on Living Lab training and capacity building.

If applicable, is the statistical analysis and its interpretation appropriate?

I cannot comment. A qualified statistician is required.

Is the study design appropriate and is the work technically sound?

Yes

Is the work clearly and accurately presented and does it engage with the current literature?

Yes

Are the conclusions drawn adequately supported by the results?

Yes

Are sufficient details of methods and analysis provided to allow replication by others?

Partly

Are all the source data and materials underlying the results available?

Partly

Reviewer Expertise:

food design, design, participatory approach, adult education, living labs, design thinking, facilitation & transdisciplinarity

I confirm that I have read this submission and believe that I have an appropriate level of expertise to confirm that it is of an acceptable scientific standard, however I have significant reservations, as outlined above.

## References

[1] : Co-creativity in Living Labs: fostering creativity in co-creation processes to transform food systems. *Journal of Science Communication* .2023;22(03) : 10.22323/2.22030203 10.22323/2.22030203

# Response

**Published:** 2026-04-08

**Authors:** Nele De Witte

**Affiliations:** Centre of Expertise Care and Well-being, Thomas More University of Applied Sciences, Geel, Belgium

Dear Dr. Massari; Thank you very much for your thorough reading of the manuscript and your suggestions. We have made the necessary changes to the manuscript and have provided a point-by-reply below.  

**Reviewer: **The manuscript presents and evaluates a modular training programme, defined as *fast-track* due to its limited duration, developed within the H2020 VITALISE project to support external researchers in accessing transnational Living Lab (LL) infrastructures in the *Health & Wellbeing* domain.

 The contribution is practical, well contextualised, and provides useful documentation of a capacity-building initiative aimed at methodological harmonisation and adoption across Living Labs. However, the evaluation framework is largely perceptual in nature (satisfaction, perceived usefulness, post-training self-efficacy) and does not fully support some of the implicit inferences present in the framing of the paper (e.g. “accelerating expertise”, “increased confidence”), which should be more carefully aligned with the limits of the available evidence.

 At the same time, it is important to underline that the paper addresses a relevant and underexplored gap in the literature, namely the training and capacity building of professionals—particularly researchers—working within Living Lab contexts. From this perspective, the manuscript represents an interesting milestone in the emerging body of literature concerned with methodologies and methods in Living Lab research and practice.

 The paper effectively highlights a real and well-recognised issue: the gap between the increasing availability of Living Lab infrastructures and the competencies required to use them in a robust, reflective, and sustainable manner. The conceptual framing that links Living Labs, co-creation, Responsible Research and Innovation (RRI), and the need for dedicated training is convincing and well grounded.

 The structure of the programme is presented through a clear sequential logic (Figure 1) and a detailed description of the training blocks (Figure 2), including welcoming and icebreaker activities, an introduction to Living Labs and ethics, VITALISE and harmonisation, methodology (theory), a methodology workshop, panel management, and an optional open block. The modular and adaptable nature of the programme represents a clear strength, particularly in relation to heterogeneous contexts and hosting Living Labs.

 The open orientation of the initiative is also commendable. The availability of public training materials, datasets, and extended resources enhances the replicability and transferability of the programme.

 Quantitative results report very high levels of satisfaction (mean 9.11/10), high perceived quality and usefulness across training blocks (approximately 4.2–4.8/5), and high post-training confidence (8.54/10). Qualitative feedback further emphasises strengths such as interaction with experts, learning efficiency, and flexibility of the format.

**Response: **Thank you for your kind words and appreciation for our endeavor to design and evaluate a training approach for living lab methodologies.  

**Reviewer:** Critical issues and recommended improvements:

 The manuscript is supported by a rich and generally well-organised body of literature that provides a clear introduction to Living Labs, wicked problems, and the adaptability of the Living Lab model to contemporary societal challenges characterised by situated, real-life, and multi-actor conditions. This framing effectively prepares the reader for the proposed intervention.

 However, I would expect a more substantial engagement with the scientific literature on competencies and capacity building, particularly with regard to the evolution of roles, skills, and professional profiles required within Living Labs. This discussion could usefully address capacity building from the perspective of the different actors involved (e.g. researchers, facilitators, managers, institutional partners), rather than remaining primarily implicit.

**Response: **We have now elaborated the introduction to inform on experiential learning and to highlight more why capacity building is needed for professionals (since for many individuals operating in the healthcare field, this was insufficiently addressed in their education).

**Reviewer: **The title and several passages in the manuscript suggest an effect in terms of *“accelerating expertise”* and/or increasing confidence or competence. However, the evaluation relies primarily on:

- satisfaction measures,
- perceived quality and usefulness,
- post-training confidence only (without a pre-training baseline),
- open-ended qualitative feedback.

In its current form, the evaluation design allows the authors to state that the training is well accepted, perceived as useful, and associated with high self-reported confidence after completion. It does not, however, support causal claims regarding an *increase* in competence, nor does it justify references to *expertise* in a strong sense (i.e. skill acquisition or demonstrable capability development).

 For this reason, a systematic revision of the language used across the title, abstract, and discussion is recommended. Alternative formulations such as *onboarding effectiveness*, *acceptability*, *perceived readiness*, *perceived competence*, or *self-reported post-training confidence* would more accurately reflect what is empirically measured.

**Response:** We have now added to the limitations section that: “The study did not employ a formal pre–post design and relied on self-reported post-training perceptions, limiting conclusions about learning gains. Consequently, the findings should be interpreted as evidence of perceived usefulness, acceptability, and feasibility rather than validation of accelerating expertise or increasing confidence.” Additionally, we have made changes to the title (now: A Fast-Track Training Program for Living Lab Methodologies: program components, acceptability, and participant perceptions) and in different manuscript sections to include terms such as ‘perceived’ and ‘self-report’.

**Reviewer: **The programme is explicitly designed to be adaptable: not all participants receive all training blocks, and some modules may be abbreviated or delivered online. While this flexibility is a clear operational strength, it introduces methodological challenges that are not sufficiently addressed in the current reporting.

 Key details that are currently missing include:

- which training blocks were delivered to which participants or sites,
- actual duration of each block in practice,
- delivery mode (in-person vs online),
- number of responses per block and overlap among respondents.

To improve interpretability, I strongly recommend the inclusion of a summary table (for instance organised by site or participant profile) detailing training exposure, delivery mode, approximate duration, and corresponding sample sizes.

**Response:** Study participants were not a homogenous group but had varying backgrounds and levels of previous exposure to living lab research. Therefore, we indeed allowed for flexibility in program administration to help tailor the program to participants’ needs. This flexibility could range from small sections being skipped or provided online to providing new content in the free block (e.g., business model preparations). However, no rigorous implementation documentation framework was adopted so we do not have exact statistics on program content presented to each participant. We acknowledge that this is a limitation and have added: “ However, while variation in implementation limited precise assessment of exposure, it reflects real-world practice, and positive perceptions across sites suggest the program’s acceptability across diverse conditions.”

**Reviewer: **There is a substantial difference between the number of respondents to the expectations survey (N = 91) and the post-training evaluation (N = 49). It is both plausible and methodologically relevant that more satisfied participants are more likely to complete the evaluation (self-selection), and that the free-of-charge nature of transnational access may further amplify positive responses.

 While the manuscript partially acknowledges this issue, it would benefit from a more explicit treatment. I suggest including a participant flow diagram or structured graphical presentation of the participation pathway (invited → pre-survey respondents → training participants → post-survey respondents). Where possible, a minimal comparison between respondents and non-respondents (e.g. by domain, site, country, or seniority) would further strengthen transparency and allow readers to better assess potential biases.

**Response: **We have also updated the limitations section, which now reads: “The study used a small sample (not including all training participants which implies vulnerability for self-selection) and was situated within the healthcare domain. To ensure generalizability, it would be beneficial to continue training initiatives and continuously evaluate and improve the content, especially when transferring to different contexts.” Additionally, we have checked distributions between responders and non-responders and have provided a bit more context in the manuscript. In the FTT survey results – expectations section, you can now read: “A total of 102 researchers who intended to visit the living labs were invited to complete the survey before the visit and 91 participants could be included in the expectations questionnaire.” In the description of the 49 respondents of the evaluation survey, we have now added: “The relative proportions in gender and researcher’s background showed a similar distribution in the participants of the evaluation questionnaire as compared to those in the expectations questionnaire.”.

**Reviewer: **At a narrative level, the manuscript appears to oscillate between two objectives:

1. describing a replicable modular training model, and
2. demonstrating its effectiveness in building expertise.

The second objective is not fully supported by the current evaluation design. I therefore recommend making explicit that the primary contribution lies in the design and acceptability / readiness evaluation of a multi-site modular training programme, rather than in demonstrating learning outcomes or expertise development.

 Relatedly, the description of the training model remains partial. Beyond directing readers to online tools, the paper does not clearly articulate explicit learning outcomes for each module. Making these outcomes explicit would significantly enhance transferability and would also enable future, more objective forms of evaluation (e.g. rubrics, protocol assessment, or other forms of structured assessment).

**Response: **We have now updated the study aims: “This paper presents a new fast-track-training program which aimed to broaden the understanding and valorisation of Living Lab methodologies within the research community. Specifically, this study presents the content of this training program and how it was designed and implemented within the VITALISE project. Additionally, we want to present the first self-reported experiences with this program, collected through a participant survey. We have also added explicit learning objectives to the training blocks.

**Reviewer: **In addition, the diary component is conceptually interesting but remains marginal in the current manuscript. Its evaluation focuses on perceived usefulness, without reporting even minimal indicators of actual adoption (e.g. frequency of use, extent of annotation). The authors should therefore make a clearer choice: either integrate the diary as a core component, supported by appropriate usage indicators, or reposition it as an additional element within the broader training toolkit.

**Response: **NoteTheBook was an optional tool which was provided to participants. Since we did not monitor use, we disclosed it here for transparency rather than to provide elaborate insights into its use and effectiveness. We have now removed some details and included in the general usefulness section of the results as opposed to a separate section.  

**Reviewer: **As a final, non-essential but valuable improvement, the paper rightly raises the issue of the economic sustainability of Living Labs, yet provides no operational indicators related to the training itself (e.g. trainer hours, preparation effort, indicative costs). Even minimal data in this respect would lend greater credibility to the discussion of scalability and cost-effectiveness.

**Response: **We have now elaborated that section in the discussion: “Given that Living Labs have faced challenges in economic sustainability (Fauth et al., 2024; Paskaleva & Cooper, 2021), careful attention must be paid to the cost-effectiveness of the FTT program. Having the current open-access training materials available already increases the feasibility of offering training since it reduces preparation times (and according costs), which are now limited to familiarizing oneself to program slides and content and reflecting on previous experiences that a trainer might share. Implementing the current training program itself, es expected take around 2 days (with around 8 hours of lecture materials without the free block). Due to its modular and flexible approach, abbreviated formats are also possible.”

**Reviewer: **Overall, the manuscript represents a useful and publishable contribution as an *intervention description combined with an acceptability evaluation*, situated within a real transnational context. It demonstrates a good level of transparency and is supported by a convincing modular training structure. The work clearly offers added value to the field.

 However, for the manuscript to be fully suitable for publication, a number of targeted revisions are required. In particular, these concern a clearer realignment between claims and evidence, greater detail regarding variability in programme delivery, and a more explicit handling of selected reporting aspects, as outlined above.

 With these improvements, the paper would constitute a solid and methodologically sound contribution to the literature on Living Lab training and capacity building.

**Response: **Thank you for these suggestions, we believe that the integration of your feedback has significantly improved the manuscript.

# Reviewer report: Reviewer response for version 1

**DOI:** 10.21956/openreseurope.22933.r65565

**Published:** 2026-01-06

**Authors:** Francesco Santarsiero

**Affiliations:** 1 LUM Giuseppe Degennaro University, Bari, Italy

The manuscript presents the design and initial evaluation of a two day Fast Track Training program developed within the VITALISE Transnational Access activities, aimed at onboarding external researchers into Living Lab Research Infrastructures and supporting harmonisation of Living Lab practices. The intervention is described as modular and adaptable and includes six main training blocks, welcoming and icebreaker, introduction to Living Labs including services and an ethical framework, VITALISE and harmonisation, methodology theory, methodology workshop, and panel management, plus an optional open block depending on the hosting lab, delivered either during the transnational access visit or online, with the important nuance that not all blocks were delivered to all participants because of tailoring and abbreviated formats. The evaluation relies on two surveys, a pre training expectations questionnaire completed by 91 applicants that captures perceived relevance of various topics, and a post training evaluation questionnaire completed by 49 participants that measures satisfaction, perceived quality and usefulness of blocks, and self reported confidence to apply Living Lab concepts, complemented by open ended qualitative feedback. The manuscript also reports on the NoteTheBook self reflection diary platform and its perceived usefulness. The main quantitative findings show very high satisfaction around 9.11 out of 10, high perceived quality and usefulness across blocks around 4.2 to 4.8 out of 5, and high post training confidence around 8.54 out of 10, with limited differences between blocks, including one statistically significant pairwise difference suggesting that Methodology Theory was rated higher in quality than Harmonisation. Qualitative feedback highlights strengths such as interaction with trainers, efficiency, and flexibility, and suggests improvements such as more interactive tool based workshops, more practical use cases, and more time for project specific discussion.

 Overall, the paper is practically valuable and relevant because it documents a structured onboarding training linked to a real transnational infrastructure initiative, provides a clear rationale grounded in the Living Lab, responsible research and innovation, and harmonisation context, and offers transparent access to materials and data repositories. However, several claims are currently stronger than what the evaluation design can support, and targeted revisions are necessary to ensure scientific soundness and interpretive robustness.

 The most critical point is the alignment between claims and evidence. The paper’s framing and wording, including accelerating expertise and statements implying increased confidence, are not fully supported by the outcomes as presented, because the evaluation is largely based on self reported satisfaction, perceived usefulness and quality, and post only confidence, without an explicit pre training baseline for confidence or objective measures of learning and skill acquisition. Without a pre post measure or comparator, one can report high post training confidence but cannot infer an increase attributable to the training, and similarly, accelerating expertise reads as a stronger competency claim than what satisfaction and confidence data can justify. The authors can address this in a scientifically sound way by revising wording consistently across title, abstract, results, and discussion to reflect what is actually measured, for example onboarding effectiveness, acceptability, perceived readiness, or perceived competence, and by replacing increased confidence with high confidence reported after the training unless pre confidence measures exist and can be reported. If the authors want to retain a stronger learning or competency claim, they should introduce and report pre post measures, even lightweight ones such as a short concept quiz, a self efficacy scale, or a rubric based assessment of a produced protocol, and ideally include a follow up assessment, for example three to six months, focused on adoption and quality of Living Lab application.

 A second scientifically important issue concerns heterogeneity in delivery and dose. The manuscript notes that not all participants received all blocks and that the program can be tailored by hosting Living Labs and or delivered online, but the current reporting does not fully quantify which participants received which blocks, how long each block lasted in practice, and how delivery mode varied. Because ratings are aggregated and some block level sample sizes differ, the interpretation of block comparisons and overall evaluation can be confounded by site specific and format specific factors. This can be addressed by adding a training exposure table, in main text or appendix, that indicates which blocks were delivered per participant or per site, delivery mode, whether the program was full or abbreviated, approximate duration per block, and the resulting sample size for each block rating, together with a brief explanation of why sample sizes differ and what matching and overlap exists among respondents for the statistical tests.

 A third issue that must be more explicitly handled is selection and attrition or non response bias. The expectations survey includes 91 respondents whereas the post training evaluation includes 49, and the manuscript acknowledges that some individuals completed the expectations survey but did not attend or did not complete the evaluation. Given the voluntary nature of transnational access and training participation and the fact that the training was free of charge, high satisfaction and favourable evaluations are plausible but also vulnerable to self selection and social desirability effects. To strengthen scientific credibility, the authors should provide a simple participant flow accounting, invited to expectations survey, completed expectations, attended training, invited to evaluation, completed evaluation, and if basic metadata are available, compare responders and non responders on a small set of variables such as domain, country, hosting lab, and seniority to gauge potential bias. At minimum, the generalisability statement should be tightened to specify that results reflect perceptions among evaluation respondents rather than all applicants.

 A fourth issue concerns statistical reporting and interpretation. While the use of Friedman and Wilcoxon for comparing block ratings can be appropriate, the paper should clarify how missingness was handled given that not all participants rated all blocks, indicate whether multiple comparison corrections were applied in post hoc testing, and report effect sizes for the significant comparisons so readers can judge practical significance. The use of trend towards significance language for a p value of 0.07 should be treated cautiously and explicitly framed as exploratory or replaced with a descriptive interpretation.

 Finally, the forced choice question asking participants to select the least appreciated block should be more carefully framed as a relative preference measure rather than evidence of dissatisfaction, because forcing a selection guarantees that some block will be chosen even if respondents found all blocks acceptable. Beyond these must fix points, several improvements would further strengthen contribution and reproducibility. Clarifying whether the primary contribution is the replicable modular training design or the evidence of acceptability and perceived readiness across multi country Living Labs would help align expectations. Adding explicit learning objectives per block would improve transferability.

 Better integrating or de emphasising the NoteTheBook component depending on whether it is central to the intervention would improve coherence, and if it is central then providing basic adoption and usage indicators, not only perceived usefulness, would strengthen evidence. Supporting the discussion on sustainability and cost effectiveness with minimal operational indicators such as trainer hours, contact hours, and resources required would also be consistent with the paper’s stated aims and would help readers judge scalability.

 With these revisions, especially the consistent alignment of claims to measured outcomes, clearer reporting of delivery heterogeneity and participant flow, and strengthened statistical transparency, the manuscript would provide a solid and scientifically defensible contribution as an intervention description and acceptability or perceived readiness evaluation for fast onboarding into Living Lab methodologies.

If applicable, is the statistical analysis and its interpretation appropriate?

Partly

Is the study design appropriate and is the work technically sound?

Yes

Is the work clearly and accurately presented and does it engage with the current literature?

Yes

Are the conclusions drawn adequately supported by the results?

Yes

Are sufficient details of methods and analysis provided to allow replication by others?

Partly

Are all the source data and materials underlying the results available?

Partly

Reviewer Expertise:

innovation management; open innovation labs; knowledge management; digital transformation

I confirm that I have read this submission and believe that I have an appropriate level of expertise to confirm that it is of an acceptable scientific standard, however I have significant reservations, as outlined above.

## References

[1] : Open Innovation Labs.2024; 10.1007/978-3-031-75533-0 10.1007/978-3-031-75533-0

# Response

**Published:** 2026-04-08

**Authors:** Nele De Witte

**Affiliations:** Centre of Expertise Care and Well-being, Thomas More University of Applied Sciences, Geel, Belgium

Dear Dr., Santarsiero; Thank you very much for your thorough reading of the manuscript and your suggestions. We have made the necessary changes to the manuscript and have provided a point-by-reply below.

**Reviewer:** The manuscript presents the design and initial evaluation of a two day Fast Track Training program developed within the VITALISE Transnational Access activities, aimed at onboarding external researchers into Living Lab Research Infrastructures and supporting harmonisation of Living Lab practices. The intervention is described as modular and adaptable and includes six main training blocks, welcoming and icebreaker, introduction to Living Labs including services and an ethical framework, VITALISE and harmonisation, methodology theory, methodology workshop, and panel management, plus an optional open block depending on the hosting lab, delivered either during the transnational access visit or online, with the important nuance that not all blocks were delivered to all participants because of tailoring and abbreviated formats. The evaluation relies on two surveys, a pre training expectations questionnaire completed by 91 applicants that captures perceived relevance of various topics, and a post training evaluation questionnaire completed by 49 participants that measures satisfaction, perceived quality and usefulness of blocks, and self reported confidence to apply Living Lab concepts, complemented by open ended qualitative feedback. The manuscript also reports on the NoteTheBook self reflection diary platform and its perceived usefulness. The main quantitative findings show very high satisfaction around 9.11 out of 10, high perceived quality and usefulness across blocks around 4.2 to 4.8 out of 5, and high post training confidence around 8.54 out of 10, with limited differences between blocks, including one statistically significant pairwise difference suggesting that Methodology Theory was rated higher in quality than Harmonisation. Qualitative feedback highlights strengths such as interaction with trainers, efficiency, and flexibility, and suggests improvements such as more interactive tool based workshops, more practical use cases, and more time for project specific discussion.

 Overall, the paper is practically valuable and relevant because it documents a structured onboarding training linked to a real transnational infrastructure initiative, provides a clear rationale grounded in the Living Lab, responsible research and innovation, and harmonisation context, and offers transparent access to materials and data repositories.

**Response:** Thank you very much for your thorough reading and kind words

**Reviewer:** However, several claims are currently stronger than what the evaluation design can support, and targeted revisions are necessary to ensure scientific soundness and interpretive robustness.

 The most critical point is the alignment between claims and evidence. The paper’s framing and wording, including accelerating expertise and statements implying increased confidence, are not fully supported by the outcomes as presented, because the evaluation is largely based on **self reported satisfaction**, **perceived usefulness and quality**, and post only confidence, without **an explicit pre training baseline** for confidence or objective measures of learning and skill acquisition. Without a pre post measure or comparator, one can report high post training confidence but cannot infer an increase attributable to the training, and similarly, accelerating expertise reads as a stronger competency claim than what satisfaction and confidence data can justify. The authors can address this **in a scientifically sound way by revising wording consistently** across title, abstract, results, and discussion to reflect what is actually measured, for example onboarding effectiveness, acceptability, perceived readiness, or perceived competence, and by replacing increased confidence with high confidence reported after the training unless pre confidence measures exist and can be reported. If the authors want to retain a stronger learning or competency claim, they should introduce and report pre post measures, even lightweight ones such as a short concept quiz, a self efficacy scale, or a rubric based assessment of a produced protocol, and ideally include a follow up assessment, for example three to six months, focused on adoption and quality of Living Lab application.

**Response:** It is indeed correct that a pre-post measurement design would have provided stronger support for the effectiveness of the training program. Additionally, measuring formal learning gains can be complex and we have therefore indeed opted to rely on self-report information. We have now added to the limitations section that: “The study did not employ a formal pre–post design and relied on self-reported post-training perceptions, limiting conclusions about learning gains. Consequently, the findings should be interpreted as evidence of perceived usefulness, acceptability, and feasibility rather than validation of accelerating expertise or increasing confidence.”. Additionally, we have made changes to the title (now: A Fast-Track Training Program for Living Lab Methodologies: program components, acceptability, and participant perceptions) and in different manuscript sections to include terms such as ‘perceived’ and ‘self-report’ to make sure that the results were not overstated.

**Reviewer:** A second scientifically important issue concerns heterogeneity in delivery and dose. The manuscript notes that not all participants received all blocks and that the program can be tailored by hosting Living Labs and or delivered online, but the current reporting does not fully quantify which participants received which blocks, how long each block lasted in practice, and how delivery mode varied. Because ratings are aggregated and some block level sample sizes differ, the interpretation of block comparisons and overall evaluation can be confounded by site specific and format specific factors. This can be addressed by adding a training exposure table, in main text or appendix, that indicates which blocks were delivered per participant or per site, delivery mode, whether the program was full or abbreviated, approximate duration per block, and the resulting sample size for each block rating, together with a brief explanation of why sample sizes differ and what matching and overlap exists among respondents for the statistical tests.

**Response:** Study participants were not a homogenous group but had varying backgrounds and levels of previous exposure to living lab research. Therefore, we indeed allowed for flexibility in program administration to help tailor the program to participants’ needs. This flexibility could range from small sections being skipped or provided online to providing new content in the free block (e.g., business model preparations). However, no rigorous implementation documentation framework was adopted so we do not have exact statistics on program content presented to each participant. We acknowledge that this is a limitation and have added: “ However, while variation in implementation limited precise assessment of exposure, it reflects real-world practice, and positive perceptions across sites suggest the program’s acceptability across diverse conditions.”

**Reviewer: **A third issue that must be more explicitly handled is selection and attrition or non response bias. The expectations survey includes 91 respondents whereas the post training evaluation includes 49, and the manuscript acknowledges that some individuals completed the expectations survey but did not attend or did not complete the evaluation. Given the voluntary nature of transnational access and training participation and the fact that the training was free of charge, high satisfaction and favourable evaluations are plausible but also vulnerable to self selection and social desirability effects. To strengthen scientific credibility, the authors should provide a simple participant flow accounting, invited to expectations survey, completed expectations, attended training, invited to evaluation, completed evaluation, and if basic metadata are available, compare responders and non responders on a small set of variables such as domain, country, hosting lab, and seniority to gauge potential bias. At minimum, the generalisability statement should be tightened to specify that results reflect perceptions among evaluation respondents rather than all applicants.

**Response: **We have now checked distributions between responders and non-responders and have provided a bit more context in the manuscript. In the FTT survey results – expectations section, you can now read: “A total of 102 researchers who intended to visit the living labs were invited to complete the survey before the visit and 91 participants could be included in the expectations questionnaire.” In the description of the 49 respondents of the evaluation survey, we have now added: “The relative proportions in gender and researcher’s background showed a similar distribution in the participants of the evaluation questionnaire as compared to those in the expectations questionnaire.” We do not have data on seniority. Country and hosting lab are more dispersed categories so less relevant to compare. We have also updated the limitations section, which now reads: “The study used a small sample (not including all training participants which implies vulnerability for self-selection) and was situated within the healthcare domain. To ensure generalizability, it would be beneficial to continue training initiatives and continuously evaluate and improve the content, especially when transferring to different contexts.”

**Reviewer:** A fourth issue concerns statistical reporting and interpretation. While the use of Friedman and Wilcoxon for comparing block ratings can be appropriate, the paper should clarify how missingness was handled given that not all participants rated all blocks, indicate whether multiple comparison corrections were applied in post hoc testing, and report effect sizes for the significant comparisons so readers can judge practical significance. The use of trend towards significance language for a p value of 0.07 should be treated cautiously and explicitly framed as exploratory or replaced with a descriptive interpretation.

**Response: **Thank you for this observation, we have now applied bonferroni adjustment to account for multiple testing. For each analysis the sample size is indicated in the results section. The results section has been updated and the trend interpretation has been removed.

**Reviewer:** Finally, the forced choice question asking participants to select the least appreciated block should be more carefully framed as a relative preference measure rather than evidence of dissatisfaction, because forcing a selection guarantee that some block will be chosen even if respondents found all blocks acceptable.

**Response: **We have now reframed the reporting of this question and its result to reflect relative preference.

**Reviewer:** Beyond these must fix points, several improvements would further strengthen contribution and reproducibility. Clarifying whether the primary contribution is the replicable modular training design or the evidence of acceptability and perceived readiness across multi country Living Labs would help align expectations. Adding explicit learning objectives per block would improve transferability.

**Response: **We have now updated the study aims: “This paper presents a new fast-track-training program which aimed to broaden the understanding and valorisation of Living Lab methodologies within the research community. Specifically, this study presents the content of this training program and how it was designed and implemented within the VITALISE project. Additionally, we want to present the first self-reported experiences with this program, collected through a participant survey. We have also added explicit learning objectives to the training blocks.

**Reviewer:** Better integrating or de emphasising the NoteTheBook component depending on whether it is central to the intervention would improve coherence, and if it is central then providing basic adoption and usage indicators, not only perceived usefulness, would strengthen evidence.

**Response: **NoteTheBook was an optional tool which was provided to participants. Since we did not monitor use, we disclosed it here for transparency rather than to provide elaborate insights into its use and effectiveness. We have now removed some details and included in the general usefulness section of the results as opposed to a highlighting it in a separate section .

**Reviewer: **Supporting the discussion on sustainability and cost effectiveness with minimal operational indicators such as trainer hours, contact hours, and resources required would also be consistent with the paper’s stated aims and would help readers judge scalability. **Response: **We have now elaborated that section in the discussion: “Given that Living Labs have faced challenges in economic sustainability (Fauth et al., 2024; Paskaleva & Cooper, 2021), careful attention must be paid to the cost-effectiveness of the FTT program. Having the current open-access training materials available already increases the feasibility of offering training since it reduces preparation times (and according costs), which are now limited to familiarizing oneself to program slides and content and reflecting on previous experiences that a trainer might share. Implementing the current training program itself, is expected to take around 2 days (with around 8 hours of lecture materials without the free block). Due to its modular and flexible approach, abbreviated formats are also possible.”

**Reviewer:** With these revisions, especially the consistent alignment of claims to measured outcomes, clearer reporting of delivery heterogeneity and participant flow, and strengthened statistical transparency, the manuscript would provide a solid and scientifically defensible contribution as an intervention description and acceptability or perceived readiness evaluation for fast onboarding into Living Lab methodologies.

**Response: **Thank you for these suggestions, we believe that the integration of your feedback has significantly improved the manuscript.
